# Fusion of Large-Scale Genomic Knowledge and Frequency Data Computationally Prioritizes Variants in Epilepsy

**DOI:** 10.1371/journal.pgen.1003797

**Published:** 2013-09-26

**Authors:** Ian M. Campbell, Mitchell Rao, Sean D. Arredondo, Seema R. Lalani, Zhilian Xia, Sung-Hae L. Kang, Weimin Bi, Amy M. Breman, Janice L. Smith, Carlos A. Bacino, Arthur L. Beaudet, Ankita Patel, Sau Wai Cheung, James R. Lupski, Paweł Stankiewicz, Melissa B. Ramocki, Chad A. Shaw

**Affiliations:** 1 Department of Molecular and Human Genetics, Baylor College of Medicine, Houston, Texas, United States of America; 2 Baylor College of Medicine, Houston, Texas, United States of America; 3 Texas Children's Hospital, Houston, Texas, United States of America; 4 Department of Pediatrics, Baylor College of Medicine, Houston, Texas, United States of America; 5 Department of Pediatrics, Section of Pediatric Neurology and Developmental Neuroscience, Baylor College of Medicine, Houston, Texas, United States of America; Duke University, United States of America

## Abstract

Curation and interpretation of copy number variants identified by genome-wide testing is challenged by the large number of events harbored in each personal genome. Conventional determination of phenotypic relevance relies on patterns of higher frequency in affected individuals versus controls; however, an increasing amount of ascertained variation is rare or private to clans. Consequently, frequency data have less utility to resolve pathogenic from benign. One solution is disease-specific algorithms that leverage gene knowledge together with variant frequency to aid prioritization. We used large-scale resources including Gene Ontology, protein-protein interactions and other annotation systems together with a broad set of 83 genes with known associations to epilepsy to construct a pathogenicity score for the phenotype. We evaluated the score for all annotated human genes and applied Bayesian methods to combine the derived pathogenicity score with frequency information from our diagnostic laboratory. Analysis determined Bayes factors and posterior distributions for each gene. We applied our method to subjects with abnormal chromosomal microarray results and confirmed epilepsy diagnoses gathered by electronic medical record review. Genes deleted in our subjects with epilepsy had significantly higher pathogenicity scores and Bayes factors compared to subjects referred for non-neurologic indications. We also applied our scores to identify a recently validated epilepsy gene in a complex genomic region and to reveal candidate genes for epilepsy. We propose a potential use in clinical decision support for our results in the context of genome-wide screening. Our approach demonstrates the utility of integrative data in medical genomics.

## Author Summary

Improvements in sequencing and microarray technologies have increased the resolution and scope of genetic testing. As a result, millions of variations are identified in each personal genome of unrelated individuals. In the context of testing for genetic diseases, identifying the variant or variants contributing to illness among such a large number of candidates is difficult. Conventional studies to identify causative variants have relied on patterns of higher frequency in affected patients compared with individuals that are well. However, it is often the rarest variations that cause human disease, making frequency information alone less useful. Many groups have turned to computational analysis to aid in interpretation of genetic variants. Epilepsy is a disease where such tools would be useful, as only a fraction of patients with suspected genetic epilepsy have a specific genetic diagnosis. To help improve variant interpretation in epilepsy, we used computational analysis to combine knowledge about genes from large cloud information sources with mutation frequency from our diagnostic laboratory to score all genes as to how likely they are to be associated with epilepsy. We use these scores to identify possible candidate genes in epilepsy, and explore other downstream applications.

## Introduction

Interpretation of high-resolution array comparative genomic hybridization (aCGH) data is made challenging by the large number of copy number variation (CNV) events identified in each individual. Analogous problems arise in interpretation of deep sequencing data where the number of variants rapidly outstrips the capacity for manual curation. Moreover, because of the recent expansion of human populations, most variation in an individual genome is rare and restricted among family lineages, making distinction between rare and pathogenic variation difficult [1]. Given the scale of variation and the challenge of profile interpretation, a number of groups have developed and utilized computational and machine learning tools to prioritize genetic data [2], [3]. Huang and colleagues analyzed the characteristics of a group of genes and their protein products known to cause phenotypes in the haploinsufficient state and compared them to those that were repeatedly deleted in a control population of apparently healthy individuals (i.e. those haplosufficient) [4]. The differences between these groups of genes were used to develop a general quantitative model to predict whether a gene deletion is likely to be deleterious. While there is broad applicability to such a prioritization scheme, it provides little guidance to help a clinician determine whether a given deletion has a role in a specific phenotype in an individual patient. Other studies assigned genes to networks based upon particular disease phenotypes, and while useful for directing further studies, these approaches did not attempt to quantify the likelihood of a gene's appropriate assignment to a given disease trait or its propensity for actually contributing to disease [5]–[7]. Other researchers have developed a computational model that takes into account genomic structure and functional elements to predict whether a given CNV is associated with intellectual disability (ID) [8]. This algorithm represents an excellent tool, however, it does not specifically predict or rank which gene(s) within the CNV are most dosage-sensitive or likely to be relevant to the phenotype. Such specific predictions are necessary to inform clinical interpretation and to aid the development of disease-centered diagnostics. Moreover, none of these tools use comparative variant frequency information between affected phenotypes in a large database to inform the scoring and prioritization schemes.

Epilepsy is a common neurological disorder for which improved computational tools could be extremely beneficial. With over 50 million individuals affected, the prevalence of epilepsy ranges from 0.2 to 2% depending on the population studied [9]. In the United States, the overall prevalence is approximately 0.5%, with a disproportionate number of cases in infants, children, and the elderly [10]. The epilepsies are currently grouped into genetic, structural/metabolic, or unknown etiologies [11]. To date, only a fraction of patients with suspected genetic forms of epilepsy have an etiological diagnosis, meaning accurate recurrence risk, prognosis, and disease-specific surveillance and treatment information are rarely available. The lack of specific diagnoses is at least partly due to the complex inheritance, variable expressivity, and incomplete penetrance of many forms of epilepsy; although some examples of Mendelian segregation are recognized [12].

The role of CNVs in common neurological diseases has become increasingly clear, and there are well-studied CNVs that cause isolated or syndromic disorders including ID [13], autism spectrum disorders (ASDs) [14], [15], and schizophrenia [16]–[18]. Although each CNV itself is rare among individuals with a given disease, when considered as a group, structural variation of the genome is a common cause of such phenotypes. A number of well-described syndromic disorders with epilepsy are caused by CNVs, including chromosome 1p36 deletion syndrome, Angelman syndrome, and *MECP2* duplication syndrome. A number of studies testing large cohorts of individuals have demonstrated that various CNVs are associated with a wide range of epilepsy phenotypes including non-syndromic idiopathic epilepsy [19]–[22].

We hypothesized that information about individual genes gleaned from large-scale knowledge sources could be integrated into an epilepsy-specific pathogenicity score. We further hypothesized that these scores could be combined with frequency information of gene disruption among individuals with epilepsy to prioritize candidate genes and interpret variants identified in personal genomes. We used a fixed set of training genes previously published as variant in Mendelian epilepsies to determine training patterns for epilepsy genes in these high dimensional data types and subsequently developed a score matching the training set for each available gene in each knowledge source. To utilize variant frequency information together with our pathogenicity scores, we took advantage of a Bayesian approach in which the gene pathogenicity scores were used to develop informative prior probabilities for the expected increase in the frequency of variants in an epilepsy population as compared to non-neurologic controls. This statistical analysis determined Bayes factors as further scores we used to rank and prioritize genes.

We then applied these gene-level scores to characterize CNVs harbored by individuals in a well-defined cohort of subjects with epilepsy identified by electronic medical record (EMR) review, and used this analysis of CNVs to assess our pathogenicity score. We also evaluated the Bayes factors comparing the results of our epilepsy cohort to a matched cohort with non-neurologic indications, and used this method to explore a possible role of multiple genes disrupted within the genome of a single individual with epilepsy. Finally, we examined the possible utility of our scheme as a clinical decision support tool for patients undergoing genome-wide testing.

## Results

### Principles of variant prioritization

**Figure 1:**
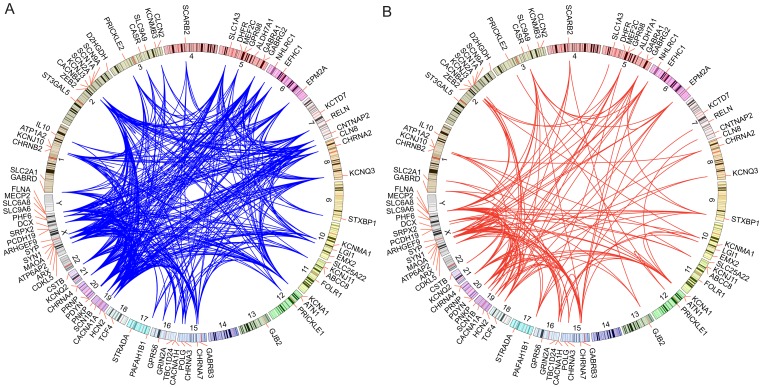
Network analysis of genes associated with epilepsy. Circos plots are drawn with the positions of a set of 83 training genes indicated along the circumference of the circles. **A.** Blue edges are drawn between genes if a given pair of genes shares an annotation to the same rare MGI knockout mouse phenotype mapped to the human orthologue. Rare annotations are defined as being annotated to 200 or fewer genes. **B.** Red edges are drawn between pairs of genes if their respective protein products physically interact or interact though exactly one intermediate interactor.

**Figure 2:**
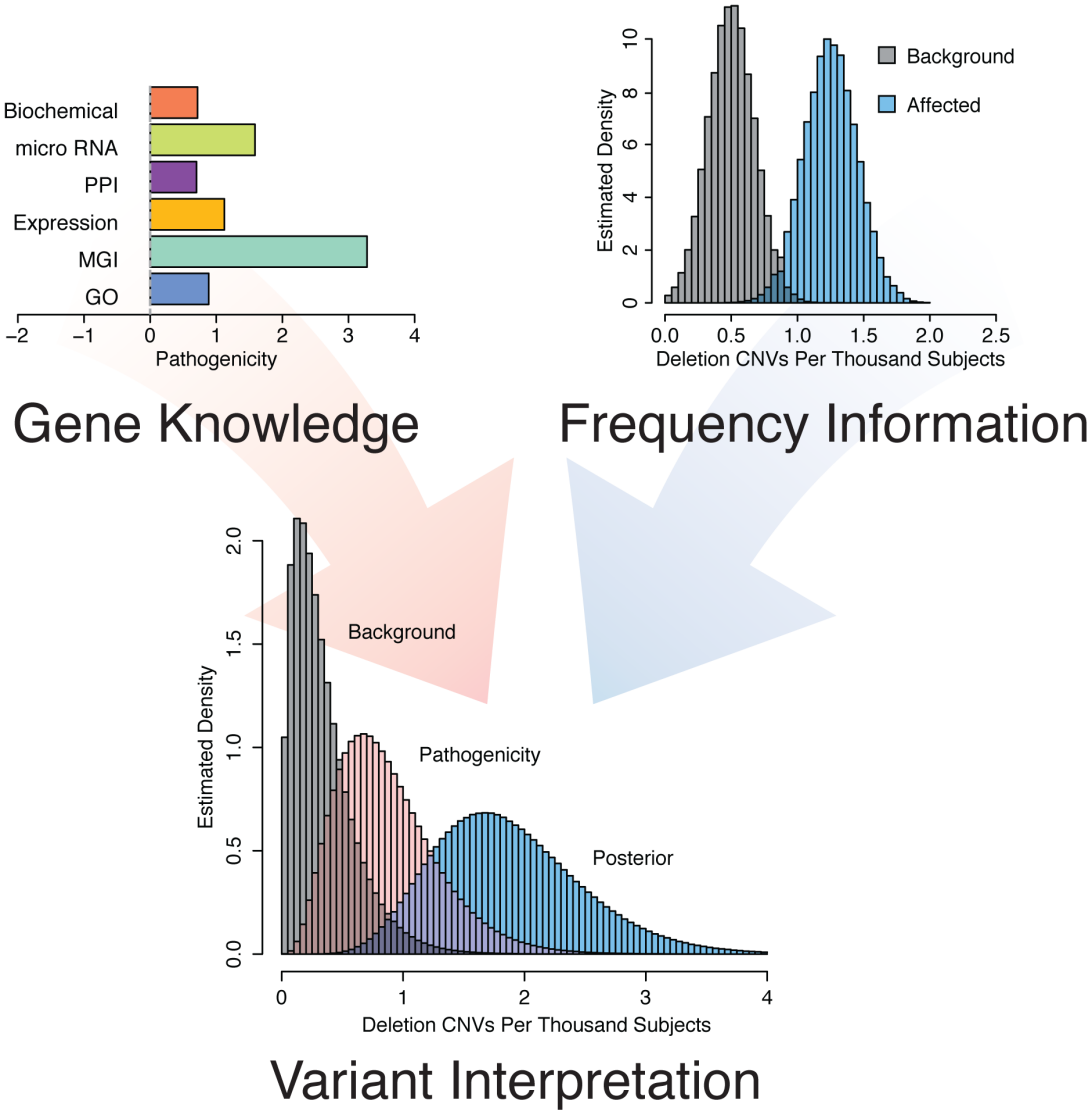
Concept of gene and variant prioritization. Top Left . Phenotype specific knowledge about each individual RefSeq gene is gained by comparing the features of the gene to a set of known “training genes” based on Gene Ontology annotation, knockout mouse phenotypes mapped to human orthologues, tissue expression patterns, protein-protein interactions, micro RNA targeting, and pathway membership information. **Top right**. Relevance to epilepsy is assessed by comparing the frequency of gene disruption among individuals with epilepsy to the frequency of disruption among a background population. **Bottom**. These two sources of information, gene knowledge and variant frequency, are combined using Bayesian methods to arrive at a Bayes factor and a posterior rate distribution to prioritize individual genes for phenotypic relevance.

Genes involved in the same disease are often similarly annotated in knowledge databases, are expressed in similar tissues or have gene products that physically interact [2]. We hypothesized that genes involved in epilepsy would show such characteristics. Indeed, analysis of a set of 83 genes with known epilepsy associations reveals that epilepsy genes form highly connected networks in multiple datasets (**Figure 1**). Thus, we concluded that it would be reasonable to interrogate these knowledge sources to identify as-of-yet unknown genes associated with the phenotype by correlating the features of the training genes with those of all other RefSeq genes. To improve our prioritization, we sought to include information about how often a given gene was mutated among individuals with epilepsy compared with a background population (**Figure 2**). These complementary strategies and their integration are described below.

### Epilepsy pathogenicity score

We hypothesized that a bioinformatic approach could consolidate information from multiple biological fields into an integrated score of pathogenicity on a genome-wide scale. To this end, we validated six “features” (see methods) using biological information from large-scale knowledge sources and comparing known epilepsy genes, including 20 recognized as causative by the International League Against Epilepsy [23] (**Table S1**), to all annotated RefSeq genes. We considered Gene Ontology (GO) and Mouse Genome Informatics (MGI) phenotype annotation, protein-protein interaction (PPI) data, human tissue expression patterns, micro RNA (miRNA) targeting, and the Kyoto Encyclopedia of Genes and Genomes (KEGG) pathway data to develop an omnibus epilepsy pathogenicity score to predict whether loss of function of a given gene might be relevant to the phenotype. To determine the efficacy of our scoring mechanism in an unbiased way, we cross-validated our approach. We removed each of the training genes and recalculated genome-wide pathogenicity scores based on the remaining training genes; we then evaluated the procedure on the gene excluded from training. The composite result is presented in **Figure S1**. The cross-validation demonstrates that individual feature scores as well as the composite mean pathogenicity score detect known epilepsy genes more efficiently than random chance. The PPI score is the most efficient feature, with an area under the curve (AUC) of 0.84. The AUC of the composite score is 0.86. The individual feature scores and composite mean pathogenicity scores of some well-known genes are presented in **Table 1**. **Table S2** provides scores for all RefSeq genes.

**Table 1:** Individual feature and composite pathogenicity scores of selected genes.

Symbol	Chr	GO	MGI	KEGG	Exp	PPI	miRNA	Mean
*BRCA1*	17	0.77	−0.48	−0.11	−0.32	−1.00	0.04	**−0.18**
*CS*	12	0.8	-	−0.11	−0.31	−0.98	0.47	**−0.03**
*C3*	5	−0.38	−0.79	0.33	1.66	−0.19	−0.64	**−0.003**
*DMD*	X	2.57	−0.96	−0.11	1.58	−0.38	−0.35	**0.39**
*ERBB4*	2	0.89	3.28	1.12	1.59	0.62	0.3	**1.30**
*MECP2*	X	−0.49	2.77	-	1.57	0.37	6.62	**1.91**
*KCNQ3*	8	5.32	3.2	-	1.24	2.00	−0.2	**2.49**
*SCN1A*	2	4.52	2.26	-	1.59	0.06	7.74	**3.23**

### Correlation of pathogenicity with epilepsy phenotype

**Figure 3:**
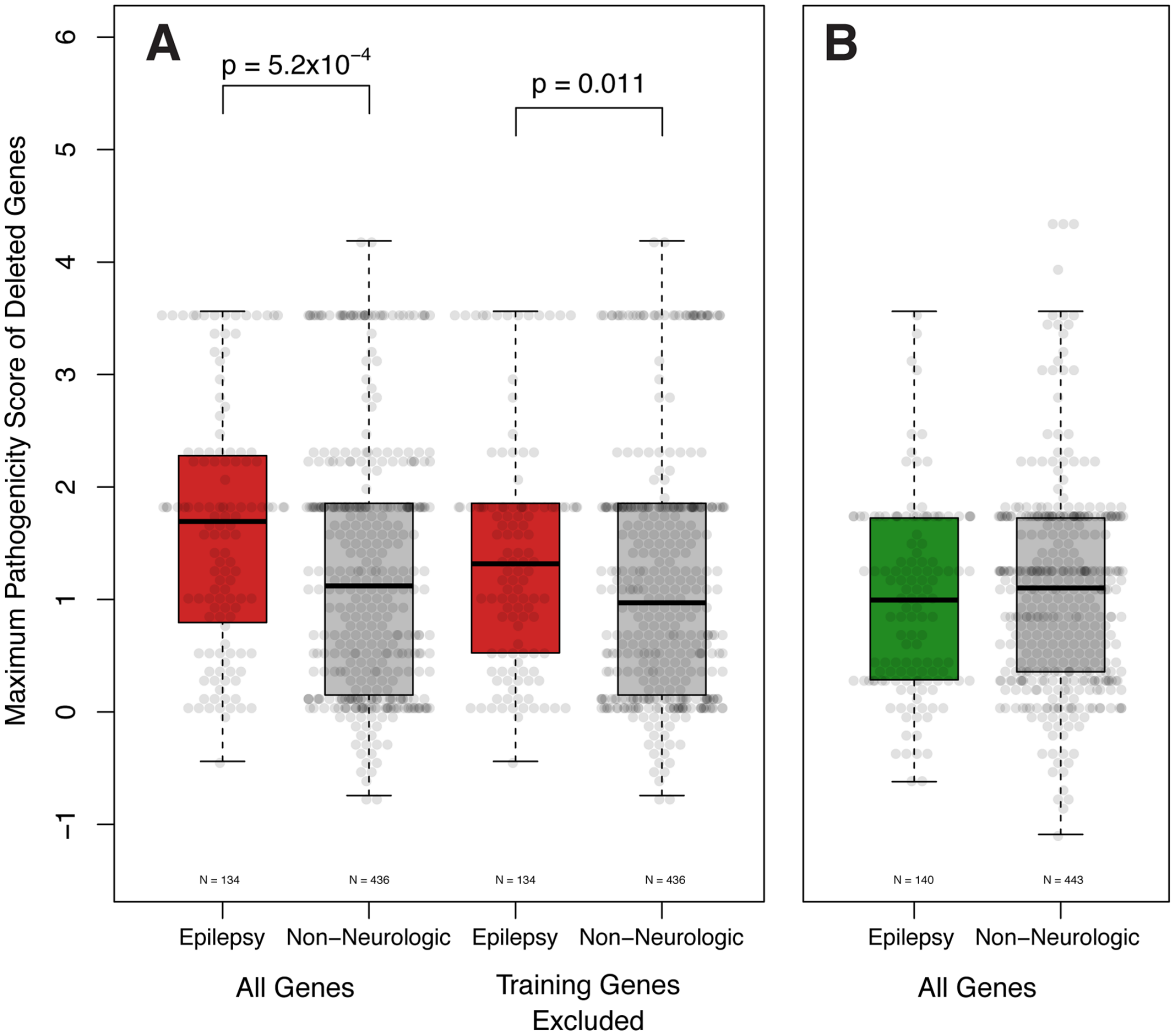
Epilepsy pathogenicity score correlates with epilepsy phenotype. A. The pathogenicity score of the highest scoring gene deleted is significantly higher in patients with epilepsy than those with non-neurologic indications (Wilcox Signed Rank Test, p = 5.2×10^−4^). This difference remains significant even when the training genes (Table S1) are not considered (Wilcox Signed Rank Test, p = 0.011). **B.** Genes contained within genomic duplications are not significantly different.

Having concluded that our pathogenicity score is capable of differentiating known epilepsy genes from other genes throughout the genome, we hypothesized that pathogenicity scores among genes deleted in individuals would correlate with an epilepsy phenotype. We theorized that patients with non-neurologic phenotypes would be less likely to harbor CNVs containing genes with high pathogenicity scores, while patients with epilepsy would have CNVs harboring genes with higher scores. To increase the likelihood of identifying an effect, we investigated the CNVs of a well-described “analysis cohort” of subjects with epilepsy identified from unbiased independent review of EMRs (see methods). We compared their CNVs to those of a matching analysis cohort of subjects referred to our diagnostic center for non-neurologic indications. We computed the maximum pathogenicity score among genes varying in copy number for each subject, filtering erroneous calls (see methods). Considering all CNVs – both gains and losses – across the two analysis cohorts, the total pathogenicity burden is not significantly different between subjects with epilepsy and subjects without neurological disease (data not shown). However, considering only genes harbored within genomic deletions reveals that the maximum scoring gene of deletion CNVs is significantly higher (Wilcox Signed Rank Test, p<5.2×10^−4^) in patients with epilepsy (**Figure 3A**). The maximum scoring genes of genomic duplications are not significantly different between the two patient groups (**Figure 3B**).

**Figure 4:**
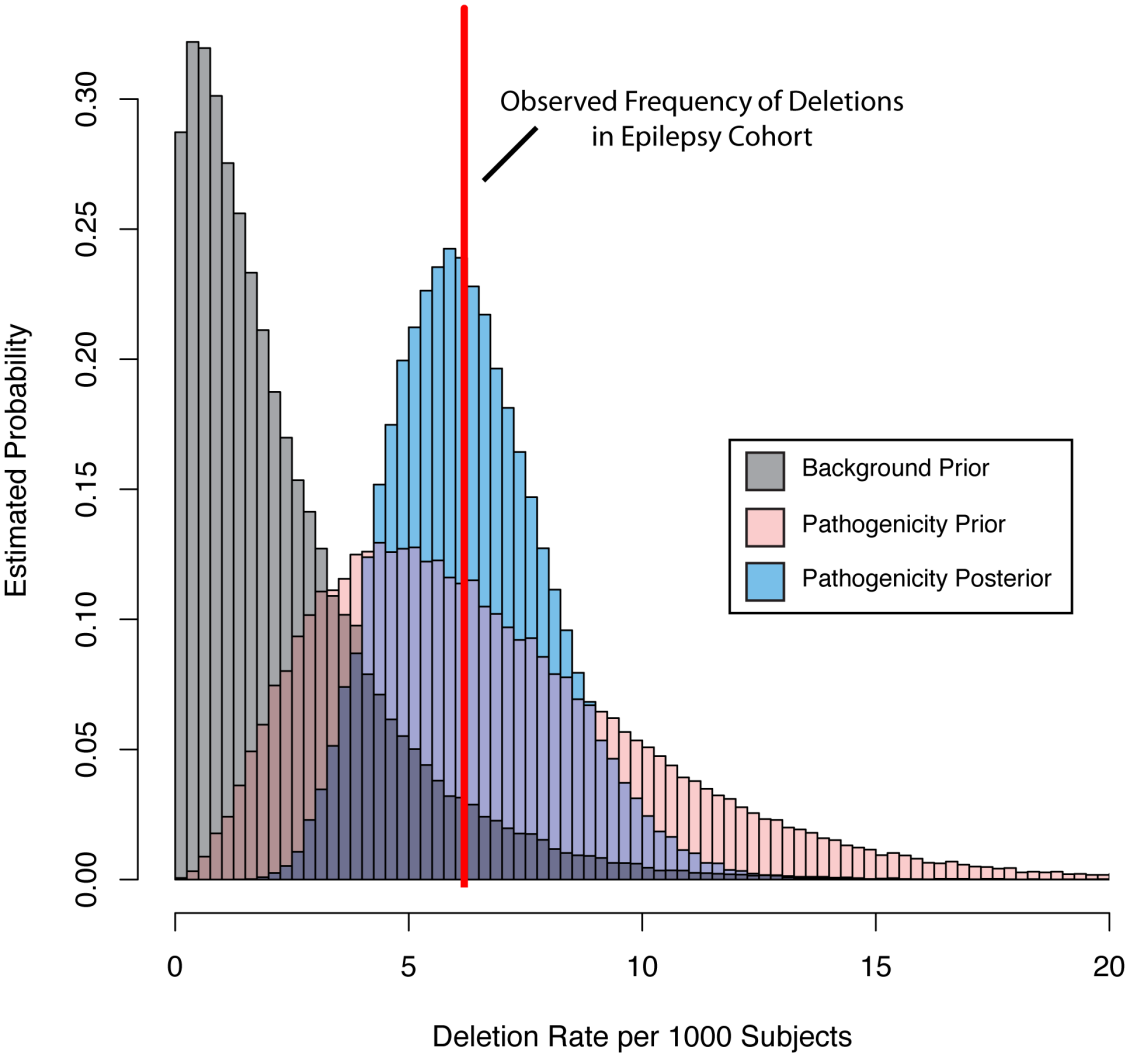
Bayesian integration of pathogenicity scores and mutation frequency for the *KCTD13* gene. We parameterized a prior gamma distribution that models the baseline deletion frequency by setting the mean equal to the observed deletion rate in a background population referred for non-neurologic indications (grey distribution). We then varied the mean of a second gamma distribution based on the pathogenicity score (pink distribution). Next, we computed the probability of the observed rate (red line) of deletions among our subjects with epilepsy under the background and pathogenicity-informed models to arrive at the Bayes factor. Finally, we determined the posterior rate distribution (blue distribution), taking into account gene knowledge from the pathogenicity score, background deletion frequency from the non-neurologic cohort, as well as the observed rate among subjects with epilepsy.

To exclude the possibility that the variation observed in patient-wide pathogenicity scores was due to known epilepsy genes, we elected to remove the training epilepsy genes (**Table S2**) from our calculations. In support of our findings, the maximum score of genes disrupted among patients with epilepsy remains statistically greater than those with non-neurologic indications (Wilcox Signed Rank Test, p<0.011, **Figure 4A**).

### Integration of gene knowledge and frequency

Having evidence that our pathogenicity score is correlated with epilepsy phenotype at the patient level, we sought to further improve our gene-based prioritization by including gene deletion frequency among a large cohort. This step is important because the pathogenicity score is uninformed about mutation or variant frequency of genes. To accomplish this integration, we took advantage of a Bayesian approach coupled with CNV data collected among 23,578 individuals referred to our diagnostic center for genome wide CNV testing because of a variety of phenotypes. We computationally identified individuals with indications for diagnostic test consistent with epilepsy (n = 1616) and those consistent with disease, but of non-neurologic etiology (n = 2940, see methods). This resulted in two matched “frequency cohorts” of individuals distinct from our well-phenotyped “analysis cohorts.” We determined the observed deletion frequency for each gene as described above.

We then parameterized a family of gamma prior distributions that modeled the baseline deletion frequency for each gene by setting the mean of the distribution equal to the observed deletion rate in the non-neurologic frequency cohort. **Figure 4** demonstrates this processes for *KCTD15*, with the baseline distribution shown in grey. We then allowed the prior mean of the gamma distribution of each gene to increase based on its epilepsy-specific pathogenicity score, while the variance was constrained to be a constant multiple of the mean. This approach resulted in a second family of prior rate distributions informed not only by gene knowledge but also background deletion frequency (**Figure 4**, pink distribution). Next, we computed the total probability of the observed rate of deletions for each gene among the 1,616 subjects in the epilepsy frequency cohort (**Figure 4**, red line) under the background model (grey distribution) and the pathogenicity informed prior (pink distribution). We calculated the ratio of these probabilities, called the Bayes factor, for each gene to allow us to further prioritize genes associated with the epilepsy phenotype. **Table 2** lists the 10 RefSeq genes with the highest Bayes factors, presenting only the maximum scoring gene for recurrent deletions with multiple high scoring genes. **Table 3** lists 10 additional genes with high Bayes factors but without known associations with epilepsy. Finally, we computed posterior rate distributions for each gene, taking into account gene knowledge from the pathogenicity score, background deletion frequency from the non-neurologic cohort as well as the observed rate in the epilepsy frequency cohort (**Figure 4**, blue distribution). **Table S2** lists the frequencies, Bayes factors and posterior rate distribution parameters for each RefSeq gene.

**Table 2:** Top ten loci ranked by Bayes factor.

Symbol	Training Gene	Known Association	Pathogenicity Score	Scaling Factor	Epilepsy Subjects	Non-Neurologic Subjects	Epilepsy Frequency (Per 1000 Subjects)	Non-Neurologic Frequency (Per 1000 Subjects)	Bayes Factor
*MECP2*	Yes	Yes	2.49	18.69	6	1	3.71	0.34	108.12
*PAFAH1B1*	Yes	Yes	3.11	24.11	5	1	3.09	0.34	70.80
*MEF2C*	Yes	Yes	2.41	18.47	3	0	1.86	0	24.14
*GABRB3*	Yes	Yes	3.36	22.12	6	3	3.71	1.02	23.79
*ERBB4*	No	No	1.30	4.47	6	0	3.71	0	19.25
*TBC1D24*	Yes	Yes	2.47	7.17	2	0	1.24	0	15.61
*KCTD13*	No	Yes	0.98	2.79	10	7	6.19	2.38	13.81
*L1CAM*	No	Yes	1.53	7.02	4	2	2.48	0.68	12.03
*CDKL5*	Yes	Yes	2.22	3.27	13	13	8.04	4.42	10.71
*SPICE1*	No	No	1.50	8.13	2	0	1.24	0	9.91

**Table 3:** Bayes factors of interesting novel candidate genes in epilepsy.

Symbol	Locus	Pathogenicity Score	Scaling Factor	Epilepsy Subjects	Non-Neurologic Subjects	Bayes Factor
*MDGA2*	14q21.3	0.81	4.39	3	1	7.52
*BHLHE22*	8q12-q13	1.82	5.22	1	0	6.62
*KCTD5*	16p13.3	0.70	5.22	2	0	4.93
*HCRTR1*	1p35.2	0.97	5.22	1	0	4.57
*DYX1C1*	15q21.3	0.43	5.22	6	0	4.45
*GRM5*	11q14	0.83	5.22	1	0	4.13
*KCTD14*	11q14.4	0.79	5.22	1	0	3.99
*GRIK4*	11q23.3	0.76	5.22	1	0	3.93
*STAC*	3p22.3	0.76	5.22	1	0	3.92
*PAX1*	20p11.22	0.75	5.22	1	0	3.87

### Epilepsy gene load among subjects with epilepsy

**Figure 5:**
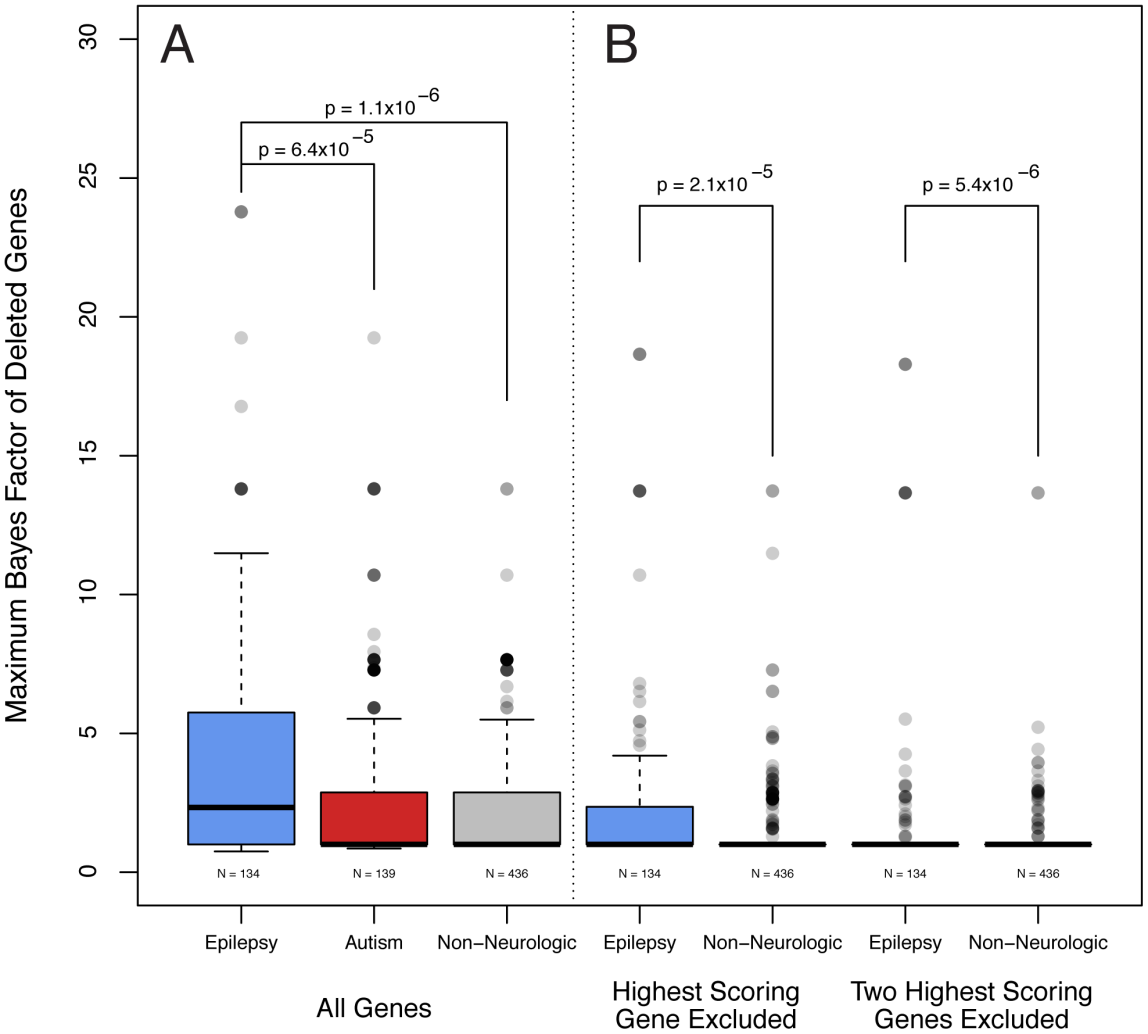
Epilepsy gene load is higher among subjects with epilepsy. A. Analogous to pathogenicity scores alone, the maximum Bayes factor of deleted genes was significantly higher among subjects with epilepsy versus non-neurologic comparators (Wilcox signed rank test, p<1.1×10^−6^). Bayes factors are also higher among subjects with epilepsy compared with those referred for autism spectrum disorders (Wilcox signed rank test, p<6.4×10^−5^). **B.** We performed the same calculation but excluded the contribution of the gene with the single highest Bayes factor from each patient. Genes with the second highest Bayes factors are significantly higher among subjects with epilepsy compared with individuals referred for non-neurologic indications (Wilxcox signed rank test, p<2.1×10^−5^). A similar test for the third highest are genes was also significant.

Having designed Bayes factors to more robustly prioritize candidate epilepsy genes, we revisited our analysis cohort of subjects with epilepsy that we previously analyzed with the pathogenicity score alone. Using a method analogous to our previous analysis performed at the level of subjects, we calculated the maximum Bayes factor among genes deleted in each individual. Because the deletion of a gene in a given subject with epilepsy necessarily influences the Bayes factor of that gene, we used a cross-validation approach, and recalculated the genome wide Bayes factors for each subject leaving out their contribution to the frequency data. We discovered that the cross-validated maximum Bayes factors were significantly higher among subjects with epilepsy than those referred for non-neurologic indications (Wilcox signed rank test p<1.1×10^−6^, p<9.4×10^−6^ without training genes **Figure 5A**). Given that many of our features preferentially identify genes that are more highly expressed in the brain (not the least of which being the gene expression feature, data not shown), we were concerned that our Bayes factors might be identifying genes associated with neurologic phenotypes rather than epilepsy in particular. To examine this, we generated a cohort of individuals referred for ASDs and not epilepsy who also had abnormal aCGH studies. In keeping with the Bayes factor score as specific for epilepsy, the maximum Bayes factor is higher among subjects with epilepsy than those referred for ASDs (Wilcox signed rank test p<6.5×10^−5^, p<2.1×10^−9^ without training genes **Figure 5A**).

We were also interested to explore whether the Bayes factors of more than one gene deleted in each individual subject might be correlated with phenotype, thus suggesting a digenic or oligogenic effect. To this end, we performed the same cross-validation calculation, but excluded the contribution of the genes with the single highest Bayes factor from each patient. Notably, the genes with the second highest Bayes factors are significantly higher among subjects with epilepsy compared with individuals referred for non-neurologic indications (Wilcox signed rank test, p<2.1×10^−5^, p<5.1×10^−5^ without training genes, **Figure 5B**). Such a comparison considering the third highest scoring gene and excluding the two highest scoring genes also results in a significant difference (Wilcox signed rank test, p<5.4×10^−6^, p<1.8×10^−6^ without training genes, **Figure 5B**).

### Assessment of pathogenicity scores and Bayes factors in a published epilepsy gene set

As additional assessment of the utility of our scoring method, we calculated the scores of genes published as potentially related to epilepsy by Lemke *et al* [24]. Because the training genes are by definition higher scoring, we elected to exclude them from this analysis. Genes in the published list but not included in the epilepsy training genes (n = 263) have significantly higher pathogenicity scores than the genome wide average (Wilcox signed rank test, p<0.014). The same genes also have significantly higher Bayes factors (Wilcox signed rank test, p<7.6×10^−12^).

### Prioritization of candidate genes at a locus with known epilepsy association

**Figure 6:**
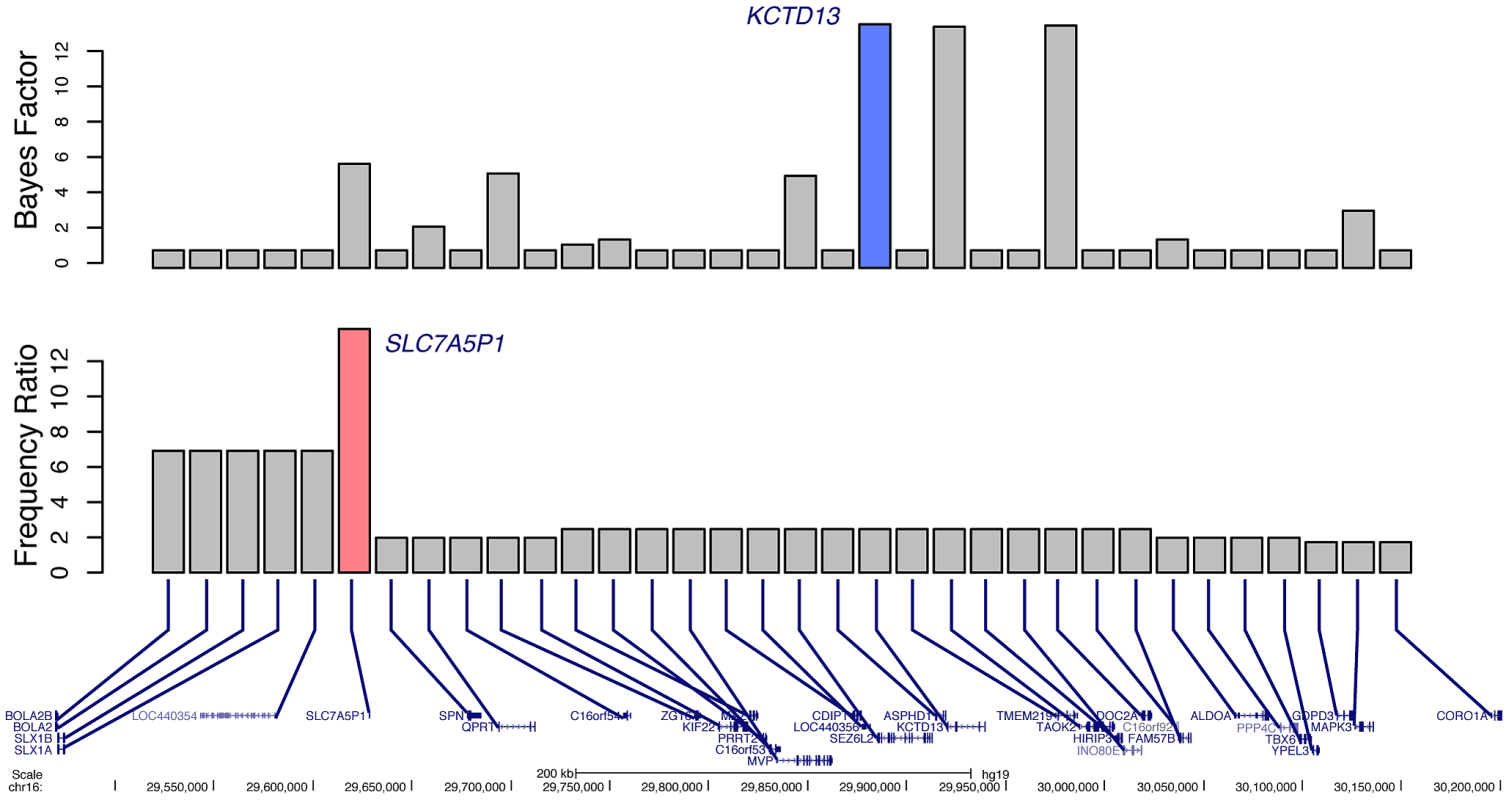
Prioritization of candidate genes at the 16p11.2 locus. Bottom . 34 RefSeq genes located within the 16p11.2 recurrent deletion. **Middle**. Ratio of deletion frequency of subjects with epilepsy to those referred for non-neurologic indications for each gene. The gene with the highest calculated ratio, *SLC7A5P1*, is highlighted in pink. **Top**. Bayes factors for each gene; the highest scoring gene, *KCTD13*, is highlighted in blue.

Another potential use of our Bayes factor scoring metric is in the identification of candidate epilepsy genes at regions with known associations but no known causative gene [25]. As a proof of principle, we analyzed the 34 RefSeq genes harbored in the recurrent, low-copy repeat mediated 16p11.2 deletion. No training gene was identified from this region because no definitive association has been made between a gene and epilepsy, although approximately 24% of patients with 16p11.2 deletions experience seizures [26]. If we use frequency data among subjects with epilepsy and those referred for non-neurologic indications alone, little information can be gained because of the recurrent nature of the deletion (**Figure 6**). In fact, because of recent advances in oligonucleotide aCGH probe design, over time additional probes have been placed in regions closer to the flanking LCRs that mediate the CNV formation. Because of this technical artifact, more subjects with epilepsy were calculated to have deletions of *SLC7A5P1* than those subjects with non-neurologic indications. However, if we instead use Bayes factors, taking into account both frequency and gene knowledge, the highest scoring gene is identified as *KCTD13* (**Figure 6**). Dosage of this gene has recently been shown to correlate reciprocally with the phenotype of head size in a Zebrafish model, a hallmark of the 16p11.2 deletion and duplication syndromes [27]. The same authors report a patient with a complex rearrangement of *KCTD13* with many of the features of 16p11.2 deletion syndrome. Nonetheless, given the high scores of both *DOC2A* and *TAOK2*, it is not unreasonable to hypothesize that one or more other genes in the region might also contribute to the epilepsy seen in these subjects. **Figure S2** presents similar analyses of other loci with recurrent deletions associated with epilepsy.

### Bayes factors to discriminate epilepsy phenotype

**Figure 7:**
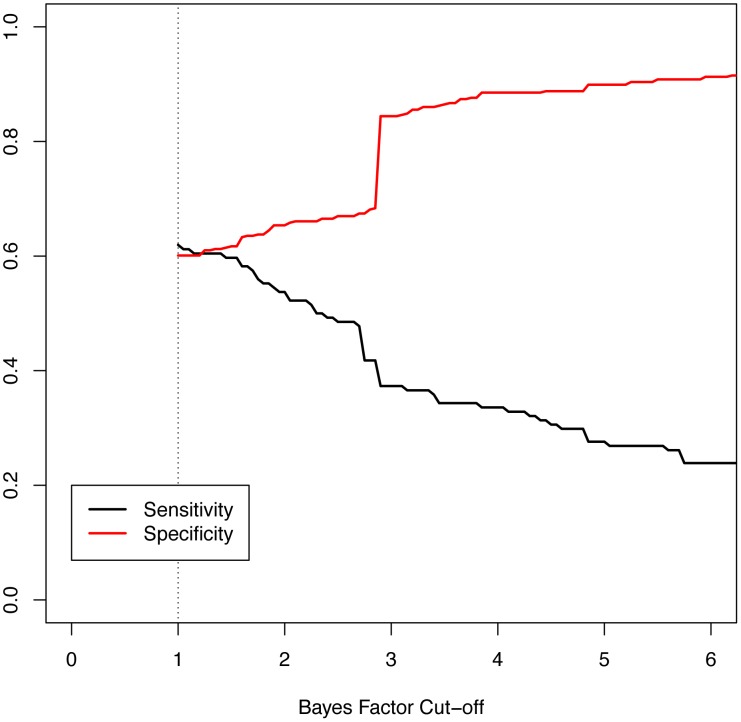
Measures of performance of the Bayes factor to discriminate epilepsy phenotype. The graphic shows the sensitivity and specificity of the Bayes factor to identify patients with epilepsy versus those referred for non-neurologic indications. The sensitivity, or the probability of having a positive test given the phenotype, is shown in black. The specificity, or the probability of a negative test given no phenotype, is shown in red. The minimum Bayes factor for a gene or patient is 1 by definition (dotted line).

Given that our data are derived from diagnostic testing, we were interested to explore our composite Bayes factor result as a possible clinical decision support tool to aid in discrimination of an epilepsy phenotype. **Figure 7** shows the sensitivity and specificity of the maximum Bayes factor among deleted genes in an individual subject when used as a binary decision rule to discriminate between epilepsy and non-neurologic phenotypes across a range of Bayes factor cut-off values. These parameters are relevant for subjects with abnormal aCGH tests and no neurologic indications other than epilepsy. As an example, having a deleted gene with a Bayes factor of greater than 1 discriminates with a sensitivity and specificity of 0.62 and 0.60, respectively. These statistics are highly influenced by recurrent deletions at the Velocardiofacial locus with a maximum Bayes factor of 2.87. Choosing a cut off of 2.88 (thus excluding the effects of the Velocardiofacial region) results in a sensitivity and specificity of 0.37 and 0.84, respectively. Given the imperfection of our indication and coding data, such a cut off rule would suggest 16% of patients with non-neurological indications should receive increased suspicion based on their CNV data. We also attempted to construct a decision rule based upon the contribution of multiple genes deleted in a given patient, but concluded the single highest scoring gene produced the best results (data not shown).

## Discussion

Rapid expansion of the human population [28] together with relaxed negative selective pressures secondary to increased food supplies and improved medical care [1] as well as the possible influence of higher mutation rates [29] have skewed much of the allele frequency spectrum of human genomic variation toward rare or private variants. Purifying selection is expected to eliminate highly deleterious alleles from a population over time [30], yet it is precisely the new and rare variations that contribute to human disease. We should expect that novel rare and private variants will continuously be discovered, and there are a nearly infinite number of possible variants and combinations of variants that can occur. Thus, a fundamental shift in the approach to variant interpretation must occur from simple cataloging of variants at a locus to prediction of the possible effects of highly rare or newly identified variants by integrating the state of knowledge about genes and disease processes. We contend that effective diagnostics must ultimately incorporate some aspects of discovery, inferring the relevance of new and arcane genomic variants for patient phenotypes by leveraging known information and multiple sources of evidence.

In essence, our approach seeks to automate aspects of expert interpretation processes that are currently undertaken by clinical molecular geneticists and diagnostic laboratories on a daily basis. These professionals consider what is known about mutated genes—for example whether they are expressed in effected tissues or if their protein products are involved in applicable pathways. They then consider the frequency of mutations both among normal individuals and patients with similar and related phenotypes. Together with other information and years of experience, the geneticist combines these data into an assessment of variant relevance. Although our computational method cannot be as effective as an experienced human at interpretation of an individual variant in a single patient, it does have the advantage of scalability to many variants and to large cohorts of individuals with different phenotypes. Moreover, this approach and others like it can help to facilitate the interpretation of an expert by providing additional triage of large-scale variant data.

Our method comprises two integrated steps: phenotype specific pathogenicity scoring and Bayesian analysis using frequency data. The pathogenicity scoring approach provides a quantitative method to evaluate genes with respect to a fixed phenotype using known phenotype specific disease genes as a target, leveraging many sources of knowledge. However, since the model depends highly on the “epilepsy genes” (**Table S1**), the choice of the training genes themselves inherently introduces the bias of past knowledge. Moreover, the training genes were not otherwise sub-structured to consider their distinct functions or roles in epilepsies with diverse etiology; this simplification was mirrored in the EMR review, where we made binary decisions about the appropriateness of the epilepsy assignment without consideration of natural history data that might otherwise inform or refine the interpretation of genetic data. Our simplified initial approach might be improved by future methods better informed by sub-classifying the training genes and refined consideration of the phenotype data.

Another facet of past knowledge bias is that the computation relies on available gene data from the literature and public databases. Thus, the pathogenicity score is only as effective as the *a priori* knowledge for each individual gene. If little or no information is known about a gene, or worse yet if a gene is not annotated in the RefSeq, the algorithm cannot accurately calculate a score. To overcome this limitation we attempted to incorporate less biased information such as gene expression data and other types of genome wide scores, such as miRNA target prediction. In cases where genes were missing features–such as lack of MGI phenotype data—we attempted to impute missing values using linear regression and other methods. Ultimately, we concluded that restricting analysis to the available reported data provided better results than statistical imputation (data not shown). More work in this area is warranted. Likewise, the knowledge sources we utilized are themselves imperfect. In ontological systems, the failure to annotate a gene to a category can represent an unobserved value in the annotation system, such as a phenotypic assay that was not performed, and not evidence of a negative annotation. This property is often summed up as: “the absence of evidence is not evidence of absence.” While this issue is an important limitation that requires further study, we believe data will improve over time, making ontological systems progressively more informative as annotations become more comprehensive genome-wide.

A key advantage of our method is incorporation of observed variant frequency data from over 20,000 genome-wide assays performed by high-resolution aCGH at out diagnostic lab in addition to the computational gene scoring approach. The epilepsy cohorts and comparator non-neurologic cohorts were comprised of phenotypically affected individuals with segmental findings. Our approach was to model the differential frequency of CNVs affecting each gene between these two groups using our pathogenicity score to inform the rate distribution. Subsequently, we are able to use the machinery of Bayesian model comparison to compute those genes where the epilepsy scoring improved the fit from what would be expected without this phenotype-based knowledge. The Bayes factor summaries allowed us to rank individual genes using the computational score, but the real frequency data—which are driven by molecular mutation events in actual human populations—necessarily incorporate structural and genomic feature information that are not part of the pathogenicity score. By exploiting variant frequency in actual subjects, our approach utilizes this genomic information without requiring us to explicitly model or otherwise include the complex biological processes underlying mutation.

Using this approach at the genome and cohort-wide level, our analysis was able to highlight a number of potentially novel genes as relevant to epilepsy. The highest scoring candidate gene identified by our method is *ERBB4*, encoding a member of the ErbB subfamily of tyrosine kinases that functions as a neuregulin receptor [31]. Rare variants of *ERBB4* have been associated with increased risk for schizophrenia [32]; an intronic deletion between exons 7 and 8 was also identified in a patient with an ASD [33]; and a patient with a *de novo* reciprocal translocation t(2;6)(q34;p25.3), apparently disrupting the *ERBB4* gene, was identified with early myoclonic encephalopathy [34]. Recent experiments also showed that *Erbb4^−/−^* mice exhibit increased susceptibility to chemically induced seizures [35]. This evidence taken together with our analysis suggests that mutations of *ERBB4* may be associated with a number of epileptic phenotypes.

Given the sizable mutation frequency difference of *ERBB4* between the epilepsy and non-neurologic cohorts, identification of the gene would have likely been possible using frequency information alone. However, our method is also able to call attention to genes that, although rarely mutated, are highly similar to the training genes. A number of the genes listed in **Table 3** exemplify this principle. For example, although *GRM5*, encoding the metabatropic glutamate receptor 5, was identified as deleted in only one subject, it's Bayes factor is in the 98^th^ percentile among genes deleted at least once in any cohort. This gene is interesting because *Grm5^−/−^* mice have increased susceptibility to pharmacologically induced seizures and the human protein product is highly connected to the epilepsy training genes. Such information could easily be overlooked given the subject's (3.6 Mb) deletion also includes 17 other RefSeq genes.

Prioritization of rarely mutated genes that are novel to the epilepsy cohort is an important aspect of our approach. Notably the exclusion of an individual's mutations from the frequency data for the Bayes factor calculation prevents contribution of such genes to our analysis cohort assessment, lowering our statistical power. Given the observation of rare variation in human disease, it is likely that some of these variants contribute to patients' seizures. Additional studies will be required to validate the associations of these genes with epilepsy.

In addition to the prioritization of individual genes, our method also naturally lends itself to calculation of multi-variant genetic load, and we were able to evaluate evidence for digenic and oligogenic effects in our analysis cohort. We saw that not only was the maximum Bayes factor among deleted genes significantly higher in the epilepsy cohort versus non-neurologic comparators, but we also observed significant differences in the second and third highest scoring genes. Previous analysis of a large set of CNV data has suggested that copy number changes of multiple genes at distant loci but in similar networks may compound at the molecular level to contribute to phenotypic variation seen with well-known recurrent genomic disorders [36]. This previous study relied on relatively more common recurrent CNVs together with second site mutations. In contrast, our method collapses different deletion alleles at the gene level and then more globally for the phenotype by scoring variants. This allows us to identify differences at the cohort level generally rather than considering individual pairs of variants for which there is very little statistical power given their rarity. Our data suggest that, at least in some patients, the deleterious effects of mutations in two or more genes involved in similar processes may interact on the molecular, cellular, or organism level to results in seizures. In other patients, genomic structural abnormalities may have little influence. Previous studies of sequence variant load in epilepsy failed to identify differences between subjects with epilepsy and control individuals [37]; however, this analysis focused entirely on channel genes whereas our method was intentionally designed to be broad and to include many gene families known to be involved in epilepsy.

Because of the strong correlation of Bayes factors with epilepsy phenotype, we investigated sensitivity and specificity of our score as a clinical decision support tool as a natural extension of our integrated analysis. Our approach was to discriminate individuals with epilepsy from those with non-neurologic indications based on their maximum Bayes factors. The difficulty experienced by physicians making use of genome wide tests represents a major limitation in clinical practice [38]. However, if genome wide results can be condensed into a quantitative score and studied epidemiologically, as are other quantitative test results (i.e. serum Troponin-I), the results may be made more accessible for physicians to interpret and guide management. For example, if a patient had a high epilepsy-specific Bayes factor but was not known to experience seizures, it might be reasonable to modify patient care. Such information could alert the clinician to provide counseling about seizure signs, symptoms, and first aid. In some instances, the prescription of an emergency abortive medication might be indicated. Neurologists are frequently asked to discern whether a patient's spells are epileptic or non-epileptic in nature; when the pre-test probability for seizures is high due to known genetic risk factors, then clinical decisions such as ordering an electroencephalogram (EEG) or longer-term video EEG monitoring or making the decision to start a medication to treat epilepsy could be impacted. While the Bayes factor score certainly does not capture the subtle differences in phenotypes caused by different alleles at various loci, this approach and extensions of it might in the future be helpful for front-line providers to identify which colleagues can provide useful insight into a particular patient's treatment or augment the clinical decision making process.

Because of its modular nature, our scoring mechanism can be recalculated at any time as more confirmed epilepsy genes are discovered, other loci throughout the genome are annotated and more fully characterized, and as additional variant frequency data are constantly recruited into our clinical genomic database. Although our model was designed to prioritize genes varying in dosage among samples tested by aCGH, the data and computational framework are not specifically tied to haploinsufficiency. Therefore, the pathogenicity scores and Bayes factors presented in **Table S2** may be applicable to a wide variety of data sets. With the decreasing cost of genome-wide sequencing strategies, analysis of patient genomes in epilepsy will likely result in an explosion of sequence variants of uncertain significance [39]. We hypothesize that together with improved algorithms designed to predict the effect of nucleotide changes on protein function, such pathogenicity scores and Bayesian methods should facilitate prioritization of sequence variants.

Overall, this study represents a step towards a quantitative framework of phenotype-specific variant interpretation. We suggest our method could be utilized for any other phenotype for which a sufficiently broad set of training genes can be generated; and although we restricted our analysis to copy number variants, this approach could be fruitfully applied to sequence variants as well. Our results have highlighted novel candidates in epilepsy and have provided further evidence of oligogenic inheritance in human disease. We believe that integrative approaches such as ours will become more accurate and useful with improved knowledge about genes and the molecular basis of disease as well as with the increased availability of genome wide profiles.

## Materials and Methods

### Epilepsy training genes

We developed a training set of clinically accepted genes that, when altered, result in epilepsy (**Table S2**), by searching the Online Mendelian Inheritance in Man (OMIM) database for all entries containing the words “epilepsy” or “seizure.” All genes matching these criteria were then manually curated based on published evidence that alterations in a given gene cause or increase susceptibility to epilepsy. Gene alterations that cause syndromes in which epilepsy is poorly penetrant, for which suspected pathogenicity is based on only a few poorly characterized patients, or for which statistically significant association was not demonstrated in at least one study were excluded. Manual review resulted in the identification of 83 epilepsy training genes and included the twenty epilepsy genes recognized by the ILAE [23]. We chose not to exclude or differentially weight genes associated with autosomal recessive or x-linked epilepsy as to have a more broadly defined pathogenicity score. This aspect is an area for further research.

### Feature design

Individual features were designed by comparing the epilepsy training gene set to every RefSeq gene based on Gene Ontology [40], MGI phenotypes [41], miRNA targeting [42], KEGG molecular interaction network data [43], GeneAtlas expression distribution [44], and PPI networks [45]. For the GO, MGI phenotypes, KEGG, and microRNA targeting data, gene level feature scores were determined by a four-step process. First, we identified annotations in each ontological system that are enriched among the set of epilepsy training genes. We used p-values for enrichment, odds ratios and number of training genes annotated to select categories; the cut-off values used to define enrichment for each annotation system are listed in **Table S3**. Second, we used a novel scoring method to quantify each gene's annotation match to the training genes. For detailed descriptions and formulae, see **Text S1**. We used these scores to determine a pair of empirical distributions, one for the training genes and one for the background genes; for each gene we computed the fraction of genes (training and all other RefSeq separately) found to have scores equal to or higher than the index gene's score. The logarithm of the ratio of these probabilities served as the metric for each gene. The log-ratio was transformed by subtracting the mean and dividing by the standard deviation and served as a standardized score for a given gene in a given annotation system. **Table S2** lists all annotation system scores for each RefSeq gene.

For gene expression we used the GeneAtlas data to identify those tissues where the epilepsy training genes were most highly expressed and where they were least expressed. Then, for each gene, we determined a score motivated by the T-statistic to measure the difference between high vs. low tissue expression. We again used the probability ratio and transformation approach to determine a score for each gene (**Table S2**).

For PPI data, we used a measure based on network communicability between each gene's protein product and those of the epilepsy training genes as our feature. Communicability measures the total number of paths that connect pairs of nodes in a network scaled by the length of each path [46]. This concept takes into account the principal that the existence or non-existence of a direct interaction between proteins does not capture fully how connected two gene products are. For example, if a pair of proteins shares a number of interacting partners, they should be considered closer than two proteins that are only connected via a single sequence of interactors of the same length. Given the scale and connectedness of the PPI network, we chose to consider all paths of length six or less (see **Text S1**). As before, we used the probability ratio and transformation approach to determine a score for each gene (**Table S2**).

Composite pathogenicity scores were generated from the mean of all 6 features. When data were missing because feature information was not present for a given gene in a given annotation system, we calculated the mean of the available features (**Table S2**). Genes for which no data was available in any feature received a zero score.

### Cross-validation of the scoring procedure

To establish the performance of our scoring procedure, we performed leave-one-out cross validation. We sequentially dropped one gene out and established the pathogenicity scores for all sources of information (GO, MGI, KEGG, miRNA, expression, and PPI) using the remaining training genes and the RefSeq complement of the full training set. Then, for the omitted training genes, we determined the individual ratios and the mean of ratios across the features. Finally, we compiled the cross-validated results and determined the percentages of epilepsy training genes that met or exceeded each percentile cutoff.

### Ethics

Approval to conduct retrospective analyses of clinical laboratory data and clinical records from the Baylor College of Medicine (BCM) Molecular Genetics Laboratory (MGL) databases and the Texas Children's Hospital (TCH) electronic medical record (EMR) using protocol H-27825 was obtained from the institutional review board for BCM and affiliated hospitals.

### Analysis cohorts

Our goal was to identify all local patients with an epilepsy diagnosis who had a clinical aCGH study performed at our institution. We searched the TCH EMR using ICD-9-CM codes 333.2, 345, 649.4, 780.39 and their respective subordinate four and five digit codes, where applicable, in order to identify patients with an epilepsy diagnosis who were seen at TCH between February 2004 and April 2011. We then identified the subset of these patients who had a clinical aCGH study in the MGL database. In an attempt to detect additional patients with epilepsy who had aCGH performed but for whom the clinician may not have properly coded the epilepsy diagnosis in the EMR, we also searched the MGL database referral indications using the search terms “epilepsy”, “seizure”, “infantile spasm”, “convulsion”, and variations thereof. In total, we identified 1,641 local patients with evidence of an epilepsy diagnosis who also had aCGH performed during the queried period of time.

Medical records for patients with abnormal aCGH test interpretations were reviewed by a diplomate of the American Board of Psychiatry and Neurology with Special Qualification in Child Neurology. Patients for whom a diagnosis of epilepsy (defined as recurrent unprovoked seizures) could not be confirmed were excluded. This resulted in a set of 295 patients with abnormal aCGH results and confirmed epilepsy. 84 of patients were tested by BAC arrays or with other manufacturers' array platforms that were incompatible with assessment across cohorts and thus were excluded, leaving 211 patient with EMR confirmed epilepsy and genome-wide results.

For comparison purposes, we also generated an analysis cohort of patients with an abnormal aCGH report from a comparable array version but no neurological referral indications or ICD-9-CM codes consistent with neurological disease. Because patients without any neurologic indication or diagnosis comprise a considerable minority of our aCGH database, we were forced to expand our search to patients referred from outside hospitals. A list of included and excluded indication and code classes are listed in **Table S4**. We also generated a comparator cohort of subjects with indications consistent with autism but not epilepsy by searching indications for the word “autism” and removing any subjects in the epilepsy cohort.

### Array CGH

Array CGH was performed on genomic DNA extracted from the patients' peripheral blood lymphocytes using various versions of oligonucleotide microarrays depending on the date of submission. Each oligonucleotide array was custom designed by the MGL at BCM (Houston, TX, USA) and manufactured by Agilent Technologies (Santa Clara, CA, USA). Data were analyzed utilizing a custom designed BCM statistical analysis package implemented in the R programming language (R Core Development Team).

### Analysis cohort pathogenicity scores

Segments of each analysis cohort individual's genome potentially varying in copy number were determined from oligonucleotide log_2_ patient vs. control intensities as previously described [11], [47]. To select calculated intervals representing true positives, we limited our analysis to intervals smaller than 15 Mb, with at least 4 variant probes, and a mean log_2_ ratio <−0.3 or >0.21. Gene lists were calculated by selecting RefSeq genes for which any part of the annotated sequence is contained within the minimum interval defined by the first and last deleted or duplicated oligonucleotide probe for each interval. The maximum composite pathogenicity score was then computed for each patient.

### Frequency cohorts

To inform our analysis using frequency information, we generated gene deletion frequency data from our clinical database. Because observed frequency rates may be influenced by changes to the microarray design, we elected to limit our analysis to 23,578 patients tested by the BCM version 8 array series. We selected individuals based on their indication for procedure as described in the analysis cohort section. This process resulted in 1,616 individuals with indications consistent with epilepsy and 2,940 individuals with indications consistent with disease but of non-neurologic etiology. These frequency cohorts fundamentally differ from the analysis cohorts in that they also contain CNVs from individuals that did not have clinically abnormal array CGH findings. Because 73 individuals in the epilepsy analysis cohort were tested by version 8 arrays, they occur in both the analysis and frequency cohorts.

### Bayesian analysis

For each RefSeq gene, we parameterized a prior gamma distribution with a mean equal to the observed rate of deletion CNVs among subjects referred to our diagnostic center for non-neurologic indications and variance constrained to be a constant multiple of the mean. If no deletion CNV was observed, we supplied a rate of 0.085 deletions per thousand subjects, equal to 1/4^th^ the lowest observed rate. We then parameterized a second gamma distribution by allowing the mean parameter to increase for genes with positive mean pathogenicity scores by scaling the mean as an increasing function of the pathogenicity score. To also take into account the rarity of the variant in question, we developed a scaling function that adjusted the influence of the pathogenicity score inversely with variant frequency in the control population (see **Text S1** and **Figure S3**). Variants commonly seen in the population would be expected to have less rate change among subjects with epilepsy. Conversely, rarely variant genes in the control population with high pathogenicity scores would be modeled to be more highly variant in epilepsy. This process resulted in two prior gamma distributions for each gene, one informed only by frequency in a non-neurologic cohort and one informed both by frequency and gene knowledge as encoded in our pathogenicity score. For genes where the pathogenicity score was negative, we retained the same prior distribution for the non-neurologic cohort. Next we calculated the probability of the observed rate of deletion CNVs among the epilepsy frequency cohort (see **Text S1**) under the two gamma distributions. The ratio of these probabilities, the Bayes factor, served as an overall score for each gene. Finally, we calculated the posterior rate parameters for each gene taking into account background frequency, pathogenicity, and the observed rate among subjects with epilepsy. **Table S2** lists the Bayes factors and posterior rate parameters for each RefSeq gene.

### Analysis cohort Bayes factors

We calculated lists of genes disrupted by deletions in each individual for all members of the autism, epilepsy and non-neurologic cohorts as described above. Because 73 members of the analysis cohort are part of the frequency cohort, their personal contribution to frequency data artificially inflates the Bayes factor score by definition. To avoid this inflation, we recalculated the genome-wide Bayes factors again, but removing their contribution to the frequency data. We then selected genes with Bayes factors greater than 1 and sorted them in decreasing order. We performed statistical analysis on the first, second, and third highest scoring genes for each individual. **Table S5** lists the top three genes and their Bayes factors for each individual.

## Supporting Information

Figure S1 Efficiency of genome-wide pathogenicity scores. Each curve represents cross validation performance (see methods) of a given feature to detect 83 known epilepsy genes. Each of the colored lines represents a single feature. The bold black line represents the composite total score based on the mean of gene ontology, MGI phenotype, pathway membership, expression, miRNA, and protein-protein interaction data. The pathogenicity score is much more efficient than random chance (dotted black diagonal line) with an AUC of 0.86.
(PDF)

Figure S2 Prioritization of candidate genes at various loci with known associations to epilepsy. Top. 1q36. Center. 2q23.1. Bottom. 16p13.11. Candidate genes with higher scoring Bayes factors are highlighted in blue.
(PDF)

Figure S3 Pathogenicity score scaling factor for low frequency variants. The graph shows the relative influence of a gene's pathogenicity score on its informative prior and Bayes factor calculation as a function of background deletion frequency. The red dashed line indicates the lowest frequency rate provided (i.e. those genes not identified as deleted in the non-neurologic population). The dashed blue line indicates the frequency of one subject with a deletion. The dashed green line indicates a frequency of 7 subjects among 2940 (2.4 per thousand subjects), the frequency of 16p11.2 deletions in our non-neurologic cohort. For a gene with a unit pathogenicity score and an observed frequency of one in the non-neurologic cohort, the scaling function determines an approximate 4.5 fold increase in rate in the epilepsy cohort.
(PDF)

Table S1 Epilepsy training genes.
(XLS)

Table S2 Pathogenicity scores, Bayes factors and posterior rate distribution parameters for each RefSeq gene.
(XLS)

Table S3 Cut-off values for determining enrichment in each annotation scheme.
(XLS)

Table S4 Included and excluded indications for the definition of a non-neurologic cohort.
(XLS)

Table S5 Genes contributing to the maximum Bayes factor analysis in each analysis cohort patient.
(XLS)

Text S1 Supplementary methods describing bioinformatic analysis.
(DOCX)

## References

[1] Lupski JR, Belmont JW, Boerwinkle E, Gibbs RA (2011) Clan genomics and the complex architecture of human disease. Cell 147: 32–43 doi:10.1016/j.cell.2011.09.008 21962505 10.1016/j.cell.2011.09.008 PMC3656718

[2] Aerts S, Lambrechts D, Maity S, Van Loo P, Coessens B, et al (2006) Gene prioritization through genomic data fusion. Nat Biotechnol 24: 537–544 doi:10.1038/nbt1203 16680138 10.1038/nbt1203

[3] Franke L, van Bakel H, Fokkens L, de Jong ED, Egmont-Petersen M, et al (2006) Reconstruction of a functional human gene network, with an application for prioritizing positional candidate genes. The American Journal of Human Genetics 78: 1011–1025 doi:10.1086/504300 16685651 10.1086/504300 PMC1474084

[4] Huang N, Lee I, Marcotte EM, Hurles ME (2010) Characterising and predicting haploinsufficiency in the human genome. PLoS Genet 6: e1001154 doi:10.1371/journal.pgen.1001154 20976243 10.1371/journal.pgen.1001154 PMC2954820

[5] Sakai Y, Shaw CA, Dawson BC, Dugas DV, Al-Mohtaseb Z, et al (2011) Protein interactome reveals converging molecular pathways among autism disorders. Science Translational Medicine 3: 86ra49 doi:10.1126/scitranslmed.3002166 10.1126/scitranslmed.3002166 PMC3169432 21653829

[6] Jia P, Ewers JM, Zhao Z (2011) Prioritization of epilepsy associated candidate genes by convergent analysis. PLoS ONE 6: e17162 doi:10.1371/journal.pone.0017162 21390307 10.1371/journal.pone.0017162 PMC3044734

[7] Kahle JJ, Gulbahce N, Shaw CA, Lim J, Hill DE, et al (2011) Comparison of an expanded ataxia interactome with patient medical records reveals a relationship between macular degeneration and ataxia. Hum Mol Genet 20: 510–527 doi:10.1093/hmg/ddq496 21078624 10.1093/hmg/ddq496 PMC3016911

[8] Hehir-Kwa JY, Wieskamp N, Webber C, Pfundt R, Brunner HG, et al (2010) Accurate distinction of pathogenic from benign CNVs in mental retardation. PLoS Comput Biol 6: e1000752 doi:10.1371/journal.pcbi.1000752 20421931 10.1371/journal.pcbi.1000752 PMC2858682

[9] Banerjee PN, Filippi D, Allen Hauser W (2009) The descriptive epidemiology of epilepsy-a review. Epilepsy Res 85: 31–45 doi:10.1016/j.eplepsyres.2009.03.003 19369037 10.1016/j.eplepsyres.2009.03.003 PMC2696575

[10] Cowan LD (2002) The epidemiology of the epilepsies in children. Ment Retard Dev Disabil Res Rev 8: 171–181 doi:10.1002/mrdd.10035 12216061 10.1002/mrdd.10035

[11] Berg AT, Berkovic SF, Brodie MJ, Buchhalter J, Cross JH, et al (2010) Revised terminology and concepts for organization of seizures and epilepsies: report of the ILAE Commission on Classification and Terminology, 2005–2009. Epilepsia 51: 676–685 doi:10.1111/j.1528-1167.2010.02522.x 20196795 10.1111/j.1528-1167.2010.02522.x

[12] Helbig I, Scheffer IE, Mulley JC, Berkovic SF (2008) Navigating the channels and beyond: unravelling the genetics of the epilepsies. Lancet Neurol 7: 231–245 doi:10.1016/S1474-4422(08)70039-5 18275925 10.1016/S1474-4422(08)70039-5

[13] de Vries BBA, Pfundt R, Leisink M, Koolen DA, Vissers LELM, et al (2005) Diagnostic genome profiling in mental retardation. Am J Hum Genet 77: 606–616 doi:10.1086/491719 16175506 10.1086/491719 PMC1275609

[14] Weiss LA, Shen Y, Korn JM, Arking DE, Miller DT, et al (2008) Association between microdeletion and microduplication at 16p11.2 and autism. N Engl J Med 358: 667–675 doi:10.1056/NEJMoa075974 18184952 10.1056/NEJMoa075974

[15] Stankiewicz P, Lupski JR (2010) Structural variation in the human genome and its role in disease. Annu Rev Med 61: 437–455 doi:10.1146/annurev-med-100708-204735 20059347 10.1146/annurev-med-100708-204735

[16] International Schizophrenia Consortium (2008) Rare chromosomal deletions and duplications increase risk of schizophrenia. Nature 455: 237–241 doi:10.1038/nature07239 18668038 10.1038/nature07239 PMC3912847

[17] Stefansson H, Rujescu D, Cichon S, Pietiläinen OPH, Ingason A, et al (2008) Large recurrent microdeletions associated with schizophrenia. Nature 455: 232–236 doi:10.1038/nature07229 18668039 10.1038/nature07229 PMC2687075

[18] Lupski JR (2008) Schizophrenia: Incriminating genomic evidence. Nature 455: 178–179 doi:10.1038/455178a 18784712 10.1038/455178a

[19] Mefford HC, Shafer N, Antonacci F, Tsai JM, Park SS, et al (2010) Copy number variation analysis in single-suture craniosynostosis: multiple rare variants including RUNX2 duplication in two cousins with metopic craniosynostosis. Am J Med Genet A 152A: 2203–2210 doi:10.1002/ajmg.a.33557 20683987 10.1002/ajmg.a.33557 PMC3104131

[20] Heinzen EL, Radtke RA, Urban TJ, Cavalleri GL, Depondt C, et al (2010) Rare deletions at 16p13.11 predispose to a diverse spectrum of sporadic epilepsy syndromes. Am J Hum Genet 86: 707–718 doi:10.1016/j.ajhg.2010.03.018 20398883 10.1016/j.ajhg.2010.03.018 PMC2869004

[21] de Kovel CGF, Trucks H, Helbig I, Mefford HC, Baker C, et al (2010) Recurrent microdeletions at 15q11.2 and 16p13.11 predispose to idiopathic generalized epilepsies. Brain 133: 23–32 doi:10.1093/brain/awp262 19843651 10.1093/brain/awp262 PMC2801323

[22] Helbig I, Mefford HC, Sharp AJ, Guipponi M, Fichera M, et al (2009) 15q13.3 microdeletions increase risk of idiopathic generalized epilepsy. Nat Genet 41: 160–162 doi:10.1038/ng.292 19136953 10.1038/ng.292 PMC3026630

[23] Ottman R, Hirose S, Jain S, Lerche H, Lopes-Cendes I, et al (2010) Genetic testing in the epilepsies–report of the ILAE Genetics Commission. Epilepsia 51: 655–670 doi:10.1111/j.1528-1167.2009.02429.x 20100225 10.1111/j.1528-1167.2009.02429.x PMC2855784

[24] Lemke JR, Riesch E, Scheurenbrand T, Schubach M, Wilhelm C, et al (2012) Targeted next generation sequencing as a diagnostic tool in epileptic disorders. Epilepsia 53: 1387–1398 doi:10.1111/j.1528-1167.2012.03516.x 22612257 10.1111/j.1528-1167.2012.03516.x

[25] Adie EA, Adams RR, Evans KL, Porteous DJ, Pickard BS (2005) Speeding disease gene discovery by sequence based candidate prioritization. BMC Bioinformatics 6: 55 doi:10.1186/1471-2105-6-55 15766383 10.1186/1471-2105-6-55 PMC1274252

[26] Zufferey F, Sherr EH, Beckmann ND, Hanson E, Maillard AM, et al (2012) A 600 kb deletion syndrome at 16p11.2 leads to energy imbalance and neuropsychiatric disorders. J Med Genet 49: 660–668 doi:10.1136/jmedgenet-2012-101203 23054248 10.1136/jmedgenet-2012-101203 PMC3494011

[27] Golzio C, Willer J, Talkowski ME, Oh EC, Taniguchi Y, et al (2012) KCTD13 is a major driver of mirrored neuroanatomical phenotypes of the 16p11.2 copy number variant. Nature 485: 363–367 doi:10.1038/nature11091 22596160 10.1038/nature11091 PMC3366115

[28] Coventry A, Bull-Otterson LM, Liu X, Clark AG, Maxwell TJ, et al (2010) Deep resequencing reveals excess rare recent variants consistent with explosive population growth. Nat Commun 1: 131 doi:10.1038/ncomms1130 21119644 10.1038/ncomms1130 PMC3060603

[29] Crow JF (2008) Maintaining evolvability. J Genet 87: 349–353. 19147924 10.1007/s12041-008-0057-8

[30] Marth GT, Yu F, Indap AR, Garimella K, Gravel S, et al (2011) The functional spectrum of low-frequency coding variation. Genome Biol 12: R84 doi:10.1186/gb-2011-12-9-r84 21917140 10.1186/gb-2011-12-9-r84 PMC3308047

[31] Anton ES, Ghashghaei HT, Weber JL, McCann C, Fischer TM, et al (2004) Receptor tyrosine kinase ErbB4 modulates neuroblast migration and placement in the adult forebrain. Nat Neurosci 7: 1319–1328 doi:10.1038/nn1345 15543145 10.1038/nn1345

[32] Law AJ, Kleinman JE, Weinberger DR, Weickert CS (2007) Disease-associated intronic variants in the ErbB4 gene are related to altered ErbB4 splice-variant expression in the brain in schizophrenia. Hum Mol Genet 16: 129–141 doi:10.1093/hmg/ddl449 17164265 10.1093/hmg/ddl449

[33] Bremer A, Giacobini M, Eriksson M, Gustavsson P, Nordin V, et al (2010) Copy number variation characteristics in subpopulations of patients with autism spectrum disorders. Am J Med Genet B Neuropsychiatr Genet 156: 115–24 doi:10.1002/ajmg.b.31142 21302340 10.1002/ajmg.b.31142

[34] Backx L, Ceulemans B, Vermeesch JR, Devriendt K, Van Esch H (2009) Early myoclonic encephalopathy caused by a disruption of the neuregulin-1 receptor ErbB4. Eur J Hum Genet 17: 378–382 doi:10.1038/ejhg.2008.180 18854870 10.1038/ejhg.2008.180 PMC2986164

[35] Li K-X, Lu Y-M, Xu Z-H, Zhang J, Zhu J-M, et al (2012) Neuregulin 1 regulates excitability of fast-spiking neurons through Kv1.1 and acts in epilepsy. Nat Neurosci 15: 267–273 doi:10.1038/nn.3006 22158511 10.1038/nn.3006

[36] Girirajan S, Rosenfeld JA, Coe BP, Parikh S, Friedman N, et al (2012) Phenotypic Heterogeneity of Genomic Disorders and Rare Copy-Number Variants. N Engl J Med 367: 1321–1331 doi:10.1056/NEJMoa1200395 22970919 10.1056/NEJMoa1200395 PMC3494411

[37] Klassen T, Davis C, Goldman A, Burgess D, Chen T, et al (2011) Exome sequencing of ion channel genes reveals complex profiles confounding personal risk assessment in epilepsy. Cell 145: 1036–1048 doi:10.1016/j.cell.2011.05.025 21703448 10.1016/j.cell.2011.05.025 PMC3131217

[38] Burke W, Emery J (2002) Genetics education for primary-care providers. Nat Rev Genet 3: 561–566 doi:10.1038/nrg845 12094234 10.1038/nrg845

[39] Gonzaga-Jauregui C, Lupski JR, Gibbs RA (2012) Human genome sequencing in health and disease. Annu Rev Med 63: 35–61 doi:10.1146/annurev-med-051010-162644 22248320 10.1146/annurev-med-051010-162644 PMC3656720

[40] Ashburner M, Ball CA, Blake JA, Botstein D, Butler H, et al (2000) Gene ontology: tool for the unification of biology. The Gene Ontology Consortium. Nat Genet 25: 25–29 doi:10.1038/75556 10802651 10.1038/75556 PMC3037419

[41] Blake JA, Bult CJ, Kadin JA, Richardson JE, Eppig JT, et al (2011) The Mouse Genome Database (MGD): premier model organism resource for mammalian genomics and genetics. Nucleic Acids Res 39: D842–D848 doi:10.1093/nar/gkq1008 21051359 10.1093/nar/gkq1008 PMC3013640

[42] Lewis BP, Burge CB, Bartel DP (2005) Conserved seed pairing, often flanked by adenosines, indicates that thousands of human genes are microRNA targets. Cell 120: 15–20 doi:10.1016/j.cell.2004.12.035 15652477 10.1016/j.cell.2004.12.035

[43] Kanehisa M, Goto S, Furumichi M, Tanabe M, Hirakawa M (2010) KEGG for representation and analysis of molecular networks involving diseases and drugs. Nucleic Acids Res 38: D355–D360 doi:10.1093/nar/gkp896 19880382 10.1093/nar/gkp896 PMC2808910

[44] Su AI, Wiltshire T, Batalov S, Lapp H, Ching KA, et al (2004) A gene atlas of the mouse and human protein-encoding transcriptomes. Proc Natl Acad Sci USA 101: 6062–6067 doi:10.1073/pnas.0400782101 15075390 10.1073/pnas.0400782101 PMC395923

[45] Cowley MJ, Pinese M, Kassahn KS, Waddell N, Pearson JV, et al (2012) PINA v2.0: mining interactome modules. Nucleic Acids Res 40: D862–D865 doi:10.1093/nar/gkr967 22067443 10.1093/nar/gkr967 PMC3244997

[46] Estrada E, Hatano N (2008) Communicability in complex networks. Phys Rev E 77: 036111 doi:10.1103/PhysRevE.77.036111 10.1103/PhysRevE.77.036111 18517465

[47] Boone PM, Bacino CA, Shaw CA, Eng PA, Hixson PM, et al (2010) Detection of clinically relevant exonic copy-number changes by array CGH. Hum Mutat 31: 1326–1342 doi:10.1002/humu.21360 20848651 10.1002/humu.21360 PMC3158569

