# Supplementary figures and images for: Fusion of Large-Scale Genomic Knowledge and Frequency Data Computationally Prioritizes Variants in Epilepsy
Source: PLoS Genet. 2013 Sep 26;9(9):e1003797. doi: 10.1371/journal.pgen.1003797 (PMC3784560; doi:10.1371/journal.pgen.1003797)

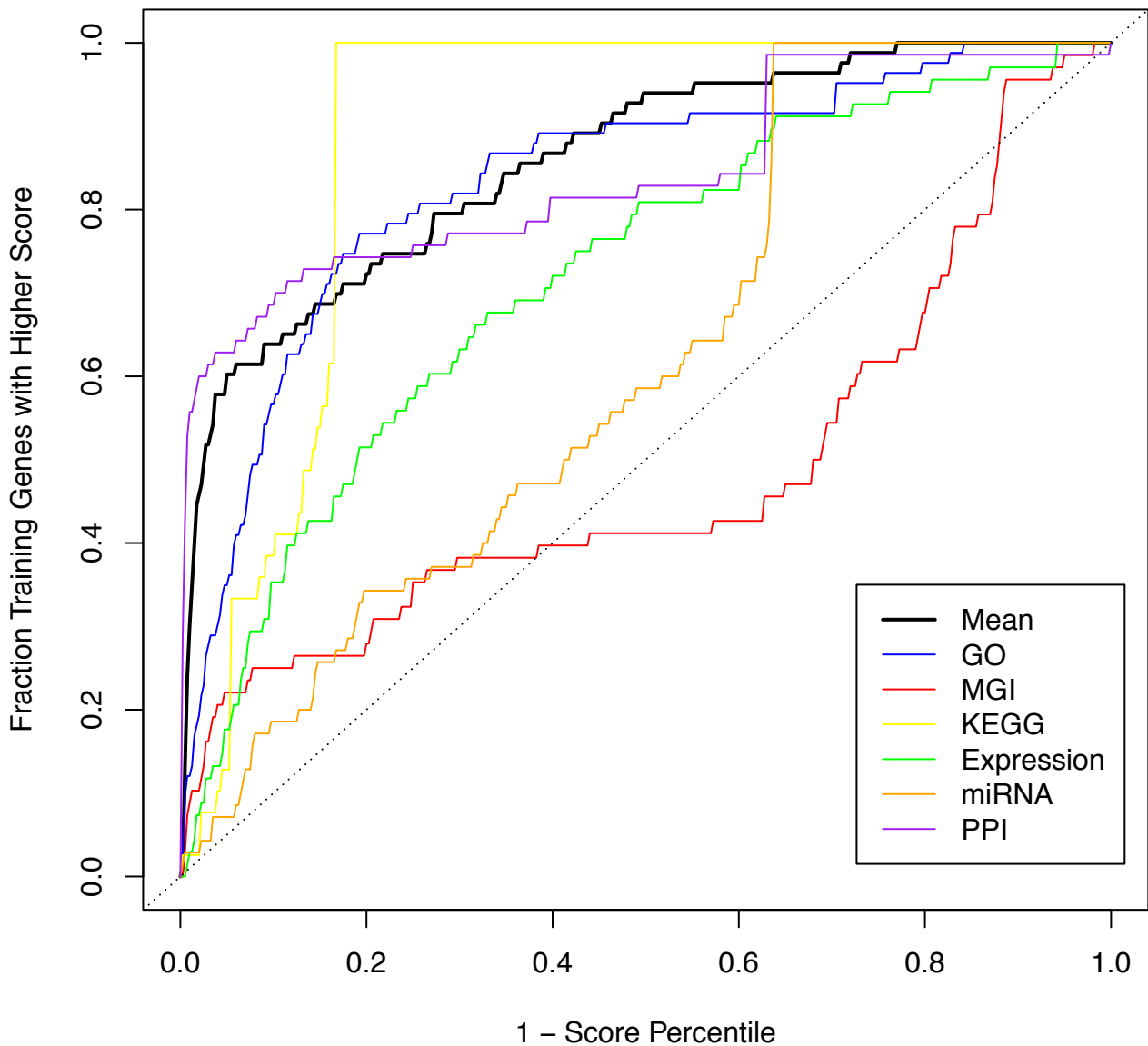

Supplement: Figure S1 — Efficiency of genome-wide pathogenicity scores. Each curve represents cross validation performance (see methods) of a given feature to detect 83 known epilepsy genes. Each of the colored lines represents a single feature. The bold black line represents the composite total score based on the mean of gene ontology, MGI phenotype, pathway membership, expression, miRNA, and protein-protein interaction data. The pathogenicity score is much more efficient than random chance (dotted black diagonal line) with an AUC of 0.86. (PDF) [file pgen.1003797.s001.pdf]

1q36

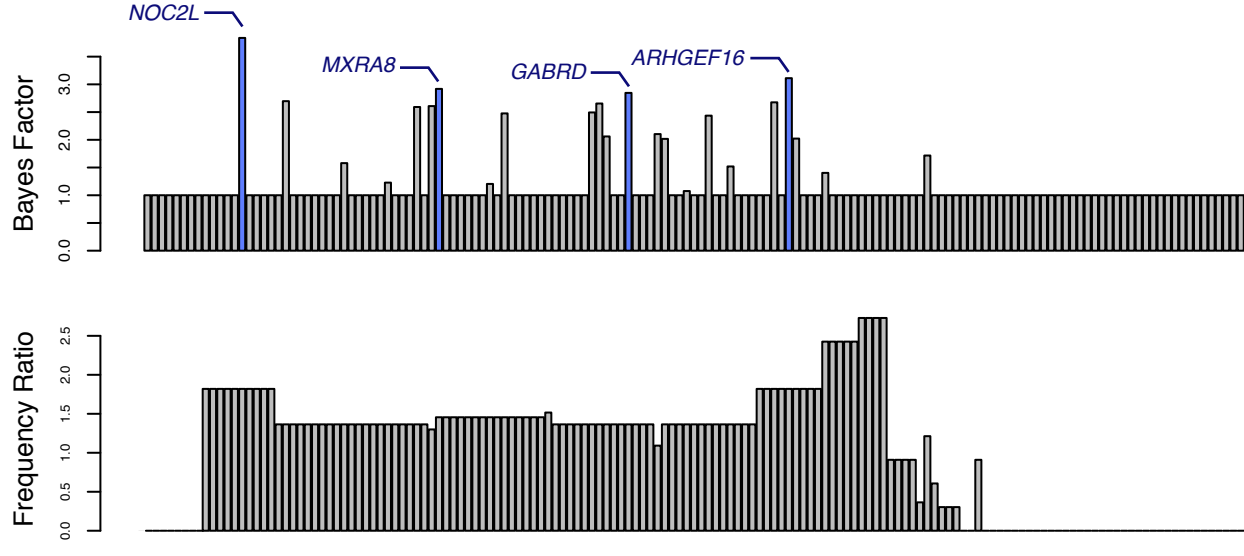

2q23.1

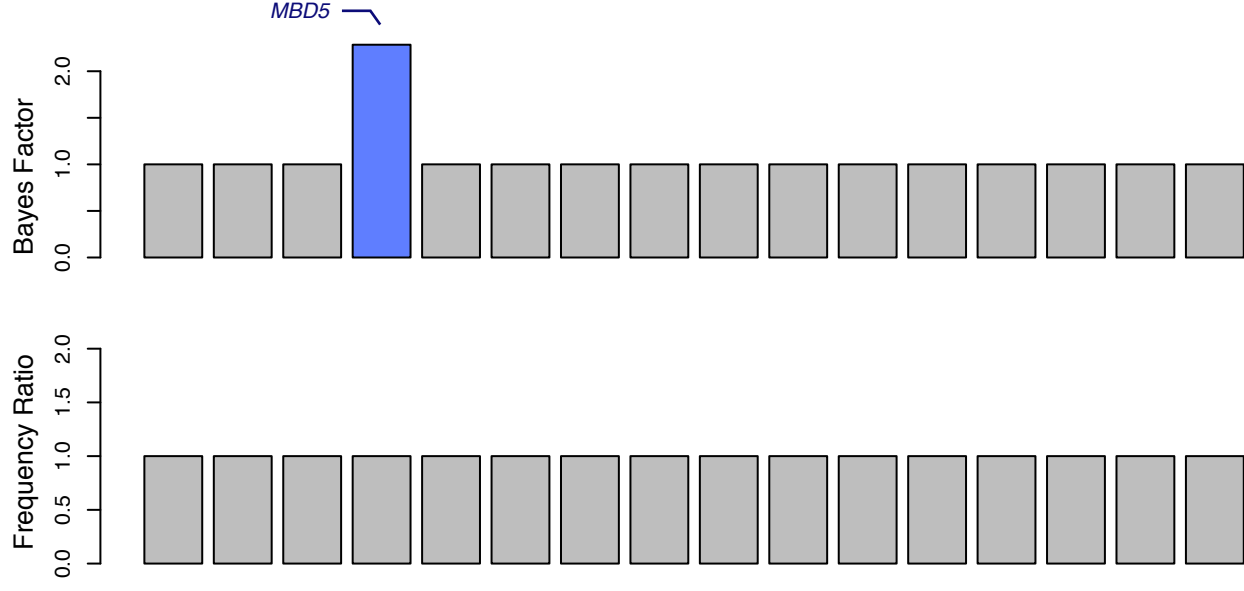

16p13.11

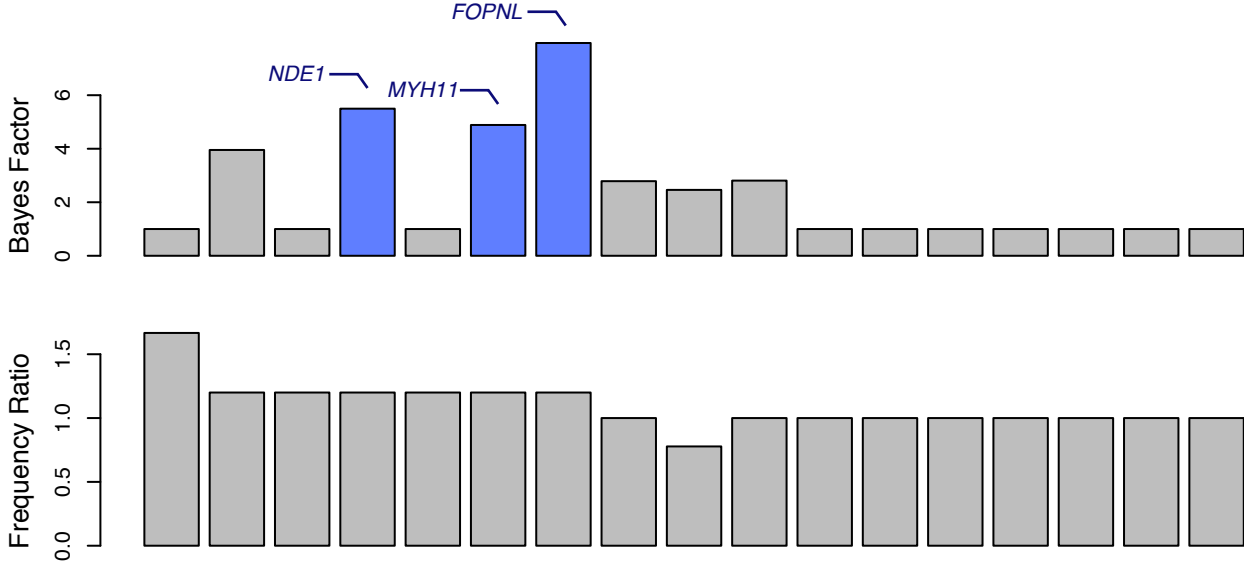

Supplement: Figure S2 — Prioritization of candidate genes at various loci with known associations to epilepsy. Top. 1q36. Center. 2q23.1. Bottom. 16p13.11. Candidate genes with higher scoring Bayes factors are highlighted in blue. (PDF) [file pgen.1003797.s002.pdf]

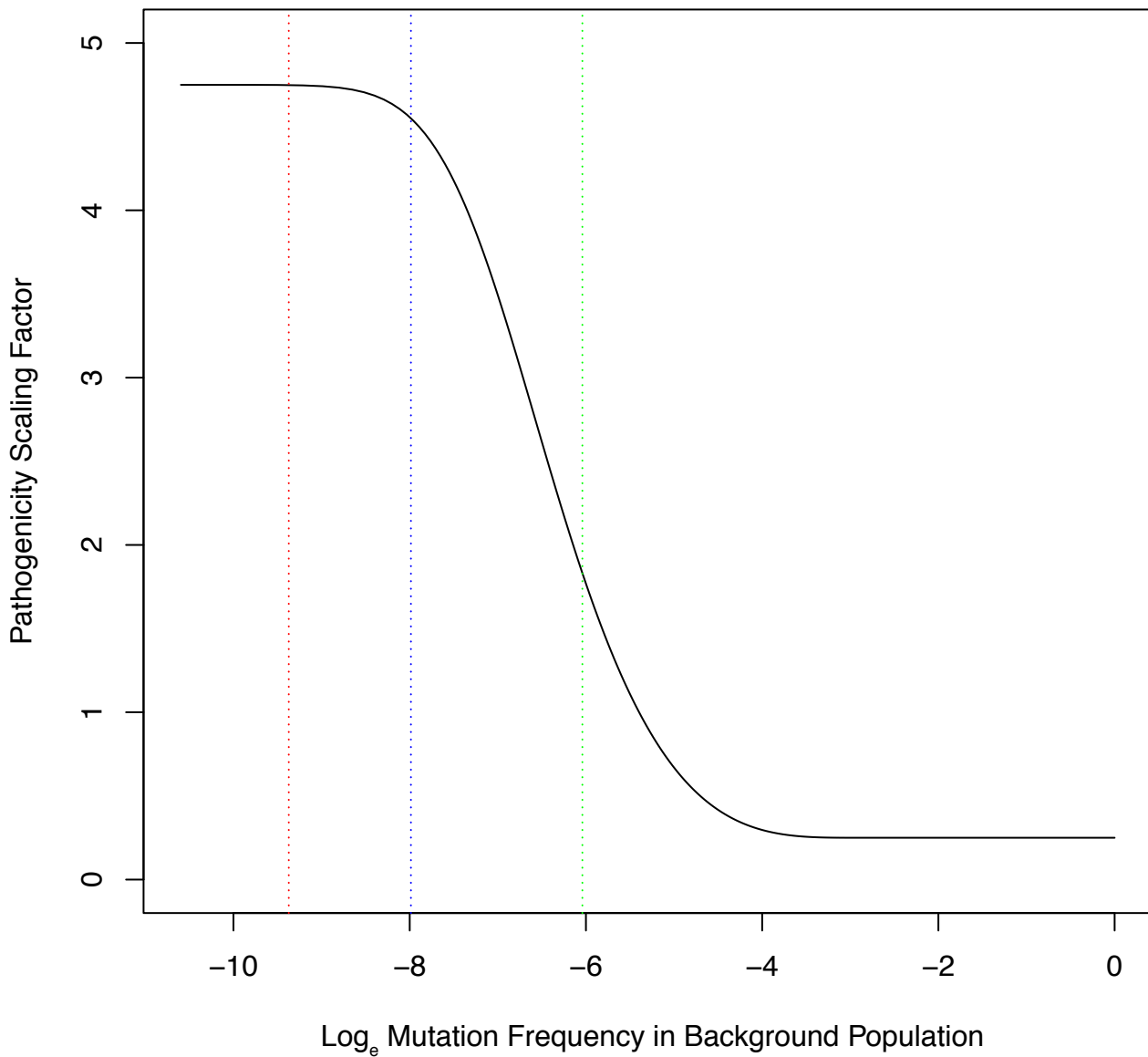

Supplement: Figure S3 — Pathogenicity score scaling factor for low frequency variants. The graph shows the relative influence of a gene's pathogenicity score on its informative prior and Bayes factor calculation as a function of background deletion frequency. The red dashed line indicates the lowest frequency rate provided (i.e. those genes not identified as deleted in the non-neurologic population). The dashed blue line indicates the frequency of one subject with a deletion. The dashed green line indicates a frequency of 7 subjects among 2940 (2.4 per thousand subjects), the frequency of 16p11.2 deletions in our non-neurologic cohort. For a gene with a unit pathogenicity score and an observed frequency of one in the non-neurologic cohort, the scaling function determines an approximate 4.5 fold increase in rate in the epilepsy cohort. (PDF) [file pgen.1003797.s003.pdf]
