# Supplementary material for: Fusion of Large-Scale Genomic Knowledge and Frequency Data Computationally Prioritizes Variants in Epilepsy
Source: PLoS Genet. 2013 Sep 26;9(9):e1003797. doi: 10.1371/journal.pgen.1003797 (PMC3784560; doi:10.1371/journal.pgen.1003797)
Supplement: Text S1 — Supplementary methods describing bioinformatic analysis. (DOCX) [file pgen.1003797.s009.docx]

*Annotation Features*

We identified categories in each annotation system that are enriched among the set of epilepsy training genes based on p-value, odds ratio, and annotated gene cutoffs (**Table S3**). Because each annotation system varies with number of annotations and number of genes assessed, we determined appropriate cut-off values based on visualization of the resultant scores’ empirical frequency distributions for the training and background genes. We used a novel method to quantify each gene’s annotation match to the training genes using the following equation:


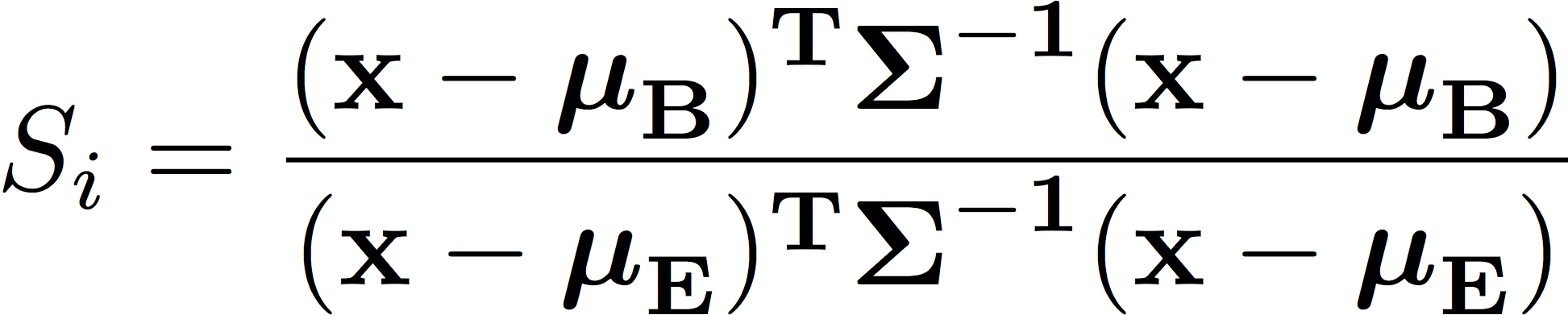


where ***x*** is a vector of annotation statuses (0 or 1) for a given gene *i* for each enriched annotation class; ***µ_E_*** and ***µ_B_*** are vectors of the expected annotation status – or frequency of the value 1 – for training genes (E) and all other genes (B), respectively, for the same annotation classes; and **Σ** is the covariance matrix of the annotation classes as determined by the random sampling model and considering all genes, but again only the annotation classes enriched among the training genes. Taking into account the covariance of annotation classes is advantageous because although two annotations may be distant in the directed acyclic graph of annotations, biological processes underlying the annotations may result in their co-annotation. For example, if a mouse has an abnormal primary motor cortex nervous system annotation, it is likely to also be annotated to abnormal motor capabilities. In this way using the covariance matrix down-weights contributions from highly synonymous annotations while emphasizing more rare categories.

Next, for each gene and set of genes (training and all other RefSeq genes separately) we computed the fraction of genes found to have scores equal to or higher than the index gene’s score. The logarithm of the ratio of these probabilities served as the metric for each gene. The log-ratio was transformed by subtracting the mean and dividing by the standard deviation of log-ratios across all genes. This standardization allowed the metrics across annotation systems to be comparable and additively combined.

*Gene Expression Feature*

For gene expression data, we used the GeneAtlas resource from BioGPS. For each gene that had multiple probe sets in this array data, we determined the probe set with the highest cross-tissue variance in gene expression as the best reporter. Then, for each probe representing a training gene we determined the expression rank of each tissue, and we determined the tissues with the 10 highest and 10 lowest median rank in expression across the training genes. The chosen top expression tissues were prefrontal cortex, cingulate cortex, amygdala, occipital lobe, medulla, pineal during night, caudate nucleus, thalamus, pons, and parietal lobe; the lowest expression tissues were CD56^+^ NK cells, B lymphoblasts, CD34^+^ hematopoietic stem cells, CD14^+^ monocytes, CD4^+^ T cells, CD8^+^ T cells, dentritic cells, CD33^+^ myeloid precursor, CD105^+^ endothelial cells, and CD19^+^ B cells. Following an approach analogous to our method for annotation content, we determined the empirical tail probabilities for T-statistic values above the value observed for both the training gene set and the background genes. We then computed and standardized the log-ratio of these empirical probabilities as our feature value for gene expression.

*Protein-Protein Interaction Feature*

For protein-protein interaction, we utilized the interactions in the PINA dataset. The complex human protein-protein interaction network can be represented as an *n* by *n* adjacency matrix whose (*i*, *j)* elements are ones or zeroes if the corresponding *i* and *j* proteins physically interact or not, respectively. A useful property of the adjacency matrix is that the (*i*, *j*) entry of the *k*^th^ power of the adjacency matrix is equal to the number of paths of length *k* starting at *i* and ending at *j*. We developed a scoring metric inspired by network communicability. For each gene we calculated a standardized score by determining the mean number of paths a given gene has to the training genes and subtracting the mean number to all other genes and dividing by the standard deviation of the number of paths to background genes. To down-weight longer paths, we scaled relative importance of each path length by dividing by the factorial of the length and summed across paths of length 1 through 6 using the equation:


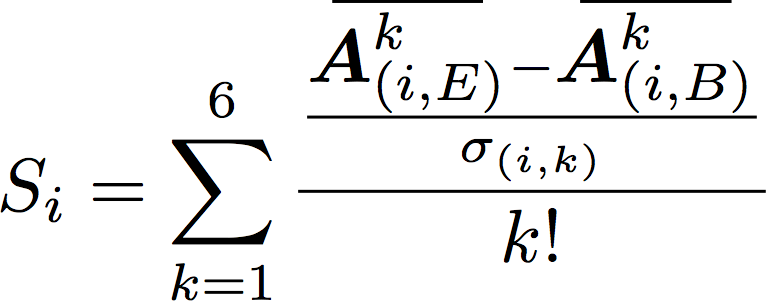


Where ***A****^k^_(i,E)_* and ***A****^k^_(i,B)_* are the row entries of the adjacency matrix raised to the *k*^th^ power for the *i*^th^ gene and column entries for the epilepsy training genes and all other genes respectively. σ is the standard deviation of the number of paths of length *k* from the *i*^th^ gene to non-training genes. We determined the empirical tail probabilities for PPI scores values observed for both the training gene set and the background genes. We then computed and standardized the log-ratio of these empirical probabilities as our feature value for protein-protein interaction.

*Composite pathogenicity score*

Standardized individual feature scores are normalized to have mean zero and variance one. Composite pathogenicity scores were generated from the mean of all 6 normalized features. When data were missing because feature information was not present for a given gene in a given annotation system, we calculated the mean of the available features. For the 4,583 RefSeq genes for which no data were available in any feature, a zero score was assigned. 55% of all RefSeq genes had data for at least half of the features.
*Bayesian analysis*

We used the Poison-Gamma conjugate family to model the CNV counts and gen-specific mutation frequencies. For each RefSeq gene we parameterized a prior gamma distribution with a mean equal to the observed rate of deletion CNVs occurring in the gene among subjects referred to our diagnostic center for non-neurologic indications and variance constrained to be two times the mean. If no deletion CNV was observed, we supplied a rate of 0.085 deletions per thousand subjects, equal to 1/4^th^ the lowest observed rate. We then parameterized a second gamma distribution by allowing the mean parameter to increase for genes with positive mean pathogenicity scores by scaling the mean as an increasing function of the composite pathogenicity score. To take into account the rarity of variation of the gene in question, we developed a scaling function that increased the influence of the pathogenicity score for lower frequency variants in the control population using the equation:


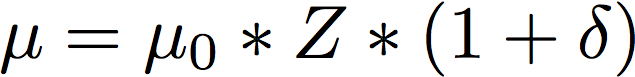


where *µ* is the adjusted mean, *µ_0_* is the observed rate in the non-neurologic cohort, *Z* is the pathogenicity score, and δ is the scaling factor. The scaling factor, δ, is given by the equation:


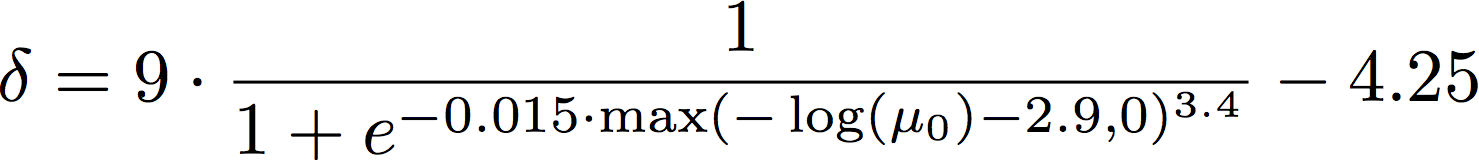


where *µ_0_* is again the observed rate in the non-neurologic cohort. **Supplementary Figure 4** shows the behavior of δ at various values of *µ_0_*. To lessen the influence of a gene with an extremely high score in a single feature, we limited the pathogenicity score to the maximum of its computed score or the number of feature for which there were data. This process resulted in two prior gamma distributions for each gene, one informed only by frequency in the non-neurologic cohort and one informed both by frequency and gene knowledge as encoded in our pathogenicity score. For genes where the pathogenicity score was negative, we retained the same prior distribution for the epilepsy cohort as used for the non-neurologic cohort. Next we calculated the observed rate of deletion CNVs among a large cohort of subjects referred to our diagnostic center for indications loosely consistent with epilepsy. We then computed a Bayes factor, *K_i_*, for each gene, *i*, using the following equation:


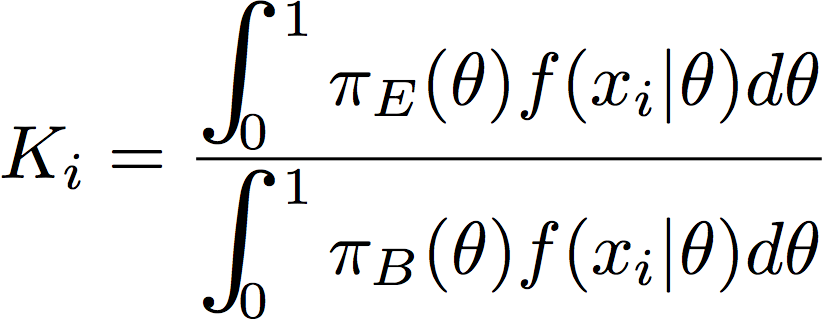


where *xi* is the observed number of subjects with epilepsy and a deletion CNV of a given gene; *θ* is the rate of deletion CNVs; π*E* and π*B* are the rate distributions under the pathogenicity (*E*) and frequency only (*B*) models, respectively; and *f*(*xi*|*θ*) is the Poison sampling distribution of *x* conditional on *θ*. Because we used the Poison-Gamma family, the Bayes factor calculation conveniently only requires evaluation of two negative binomial probabilities with parameters determined by the prior distributions. Finally, we calculated posterior Gamma rate distributions for each gene taking into account background frequency, pathogenicity, and the observed rate among subjects with epilepsy.

*Leave-one-out Bayes factor analysis*

As part of our analysis of the Bayes factor at the subject level, we computed the maximum Bayes factor among genes deleted in each subject. Because the presence of a gene deletion in a given subject with epilepsy necessarily increases the Bayes factor for genes with positive pathogenicity scores we recalculated the genome-wide Bayes factors for each subject leaving out their contribution to the frequency data. This has the unfortunate side-effect of completely excluding singleton mutations – those that are observed once in the epilepsy cohort. Nonetheless, we performed other analysis, such as those identifying novel candidates using the full data.
